# Expression of NK cluster genes in the onychophoran *Euperipatoides rowelli*: implications for the evolution of NK family genes in nephrozoans

**DOI:** 10.1186/s13227-018-0105-2

**Published:** 2018-07-18

**Authors:** Sandra Treffkorn, Laura Kahnke, Lars Hering, Georg Mayer

**Affiliations:** Department of Zoology, Institute of Biology, University of Kassel, Heinrich-Plett-Str. 40, 34132 Kassel, Germany

**Keywords:** Homeobox genes, NK genes, NK-linked genes, Gene expression, Velvet worms, Onychophora, Mesoderm development, Nephrozoa, Urbilaterian

## Abstract

**Background:**

Understanding the evolution and development of morphological traits of the last common bilaterian ancestor is a major goal of the evo-devo discipline. The reconstruction of this “urbilaterian” is mainly based on comparative studies of common molecular patterning mechanisms in recent model organisms. The NK homeobox genes are key players in many of these molecular pathways, including processes regulating mesoderm, heart and neural development. Shared features seen in the expression patterns of NK genes have been used to determine the ancestral bilaterian characters. However, the commonly used model organisms provide only a limited view on the evolution of these molecular pathways. To further investigate the ancestral roles of NK cluster genes, we analyzed their expression patterns in the onychophoran *Euperipatoides rowelli*.

**Results:**

We identified nine transcripts of NK cluster genes in *E. rowelli*, including single copies of *NK1*, *NK3*, *NK4*, *NK5*, *Msx*, *Lbx* and *Tlx*, and two copies of *NK6*. All of these genes except for *NK6.1* and *NK6.2* are expressed in different mesodermal organs and tissues in embryos of *E. rowelli*, including the anlagen of somatic musculature and the heart. Furthermore, we found distinct expression patterns of *NK3*, *NK5*, *NK6*, *Lbx* and *Msx* in the developing nervous system. The same holds true for the NKL gene *NK2.2*, which does not belong to the NK cluster but is a related gene playing a role in neural patterning. Surprisingly, *NK1*, *Msx* and *Lbx* are additionally expressed in a segment polarity-like pattern early in development—a feature that has been otherwise reported only from annelids.

**Conclusion:**

Our results indicate that the NK cluster genes were involved in mesoderm and neural development in the last common ancestor of bilaterians or at least nephrozoans (i.e., bilaterians to the exclusion of xenacoelomorphs). By comparing our data from an onychophoran to those from other bilaterians, we critically review the hypothesis of a complex “urbilaterian” with a segmented body, a pulsatile organ or heart, and a condensed mediolaterally patterned nerve cord.

**Electronic supplementary material:**

The online version of this article (10.1186/s13227-018-0105-2) contains supplementary material, which is available to authorized users.

## Background

Understanding morphological and major developmental features of the metazoan stem species is one of the major challenges of comparative developmental biology [1, 2]. In particular, the “urbilaterian” has drawn a great deal of attention and resolving the putative morphology of this hypothetical triploblastic animal has proven to be particularly challenging [1–5]. The reconstruction of the “urbilaterian” is primarily based on striking similarities of molecular patterning mechanisms and developmental pathways between extant bilaterian taxa, including the most popular model organisms like the fruit fly *Drosophila melanogaster*, the nematode *Caenorhabditis elegans*, the annelid *Platynereis dumerilii*, and the mouse *Mus musculus* [1]. These molecular pathways include the anterior–posterior patterning system involving the Hox genes [2, 6, 7], the dorsoventral patterning system using the antagonistic interaction of *sog*/*chordin* and *dpp*/*BMP2/4* signaling [8, 9], the *pax6*/*eyeless* patterning system for eye development [1, 10, 11], body segmentation processes [4, 12], the anterior patterning system involving *empty spiracles* and *orthodenticle* [13], and the posterior patterning system involving *even skipped* and *caudal* [14, 15].

Due to these genetic similarities, a complex “urbilaterian” has been proposed, which might have possessed a complex morphology including, e.g., body segmentation, a centralized nervous system, a differentiated somatic and visceral musculature, and a circulatory system with a pulsatile vessel or heart [1, 2]. However, recent phylogenetic analyses have shown that the morphologically simpler organized Xenacoelomorpha (Acoela + Nematodermatida + *Xenoturbella*) might be the sister group of all remaining bilaterians (Protostomia + Deuterostomia, or “Nephrozoa”), leading some authors to suggest a rather simple “urbilaterian” [16]. Hence, studies increasingly focus on revealing the morphological traits of the “urnephrozoan” rather than the “urbilaterian” [16, 17].

In addition to the conserved developmental genes mentioned above, the NK homeobox genes have been identified as key players in many molecular pathways that have been used to reconstruct the morphological characteristics of the “urbilaterian” or “urnephrozoan” [7, 17–19]. The NK genes were first identified in *Drosophila melanogaster* in 1989 by Kim and Niremberg [20], who established the term “NK genes” using their own initials. These genes belong to the Antennapedia class (ANTP) of homeobox genes, which is one of the largest homeobox gene classes in bilaterians, comprising the Hox, ParaHox, NK, Mega-homeobox and SuperHox gene families [7, 21]. Like the Hox and ParaHox genes, the NK genes are typically arranged in one or several clusters. However, the NK cluster shows a much higher degree of rearrangement due to extensive breakup, inversion and reunion events in different taxa [22, 23]. Based on the composition of the NK cluster in insects, combined with the information from chordates, it has been deduced that the ancestral bilaterian NK cluster comprised nine genes, namely *NK1*, *NK3*, *NK4*, *NK5*, *NK6*, *NK7*, *Msx*, *Lbx* and *Tlx* [7, 21–24]. In addition to the NK cluster genes, there is a variety of other related genes—the so-called NK-linked (NKL) genes [7]. These genes are defined as NKL genes because they show more sequence similarities to the NK cluster genes than to any other genes of the ANTP class, although they are located outside the NK cluster [7]. However, their evolutionary origin is not fully understood [7, 21].

Comparative genomic analyses and gene expression studies revealed that the NK cluster genes are expressed in mesodermal derivatives across bilaterians, including the dorsal pulsatile vessel or heart, the visceral mesoderm, and various somatic muscles [6, 23, 25–27]. For example, *NK4* is involved in the development of pulsatile organs in all bilaterians studied thus far [18, 19, 28–30]. There has been a long debate on whether or not the simple contractile vessels of invertebrates are homologous to the complex vertebrate heart. This is reflected by the existence of two different definitions of what a “heart” is [31–33]. While some authors regard only the chambered circular pumps of vertebrates as a heart, implying an anatomical homology, others use a functional definition, regarding any organ that propels fluid through a circulatory system as a heart [31, 32]. In this study, we apply the latter, more general and common definition [18, 33–36]. The striking similarities in the *NK4* expression patterns across bilaterians have led to the hypothesis that the invertebrate pulsatile vessels and the complex vertebrate heart might indeed be homologous structures [35]. Other NK cluster genes, such as *Msx* and *Lbx*, are expressed in specific sets of muscles, suggesting that they specify identity and position for the somatic musculature among bilaterians [18, 19, 37], whereas *NK3* is mainly expressed in the developing visceral mesoderm in most species studied [19, 28, 38–41]. These similarities have been used to propose a complex “urbilaterian,” which is believed to have possessed a differentiated somatic and visceral musculature as well as a pulsatile dorsal vessel [1, 19].

In addition to a function in mesoderm patterning, some NK cluster and NKL genes also show conserved expression patterns in the developing nervous system in both vertebrates and invertebrates [17–19, 42, 43]. In particular, *Msx*, *NK6* and *NK2.2* are involved in the mediolateral patterning (“dorsoventral patterning” sensu Martín-Durán et al. [17]; we favor the term “mediolateral” to avoid confusion with the dorsoventral patterning regulated by the *sog*/*dpp* system) of the trunk nervous system in both protostomes [18, 19, 44] and deuterostomes [42, 43, 45, 46]. Together with other transcription factors, these genes are expressed in a staggered mediolateral pattern in the neuroectoderm [17, 42–46]. These similarities have been used as a key argument for proposing a condensed medial ventral nerve cord for the bilaterian ancestor [43, 44]. However, this hypothesis has been challenged by a recent, more detailed analysis of the mediolateral patterning system in representatives of Xenacoelomorpha and Spiralia, which revealed that this pattern is missing in many of these taxa, indicating convergent evolution of the bilaterian nerve cords [17].

Interestingly, a study of NK genes in the annelid *Platynereis dumerilii* even revealed that some NK genes are expressed in a segment polarity pattern early in development, which has lead the authors to assume that NK genes might have been involved in segment formation in the last common bilaterian ancestor [19]. Hence, the question arises of whether or not this pattern is also found in other bilaterians.

In order to further explore the putative ancestral roles of NK cluster genes among nephrozoans, we investigated their expression patterns in embryos of the onychophoran *Euperipatoides rowelli*. Together with tardigrades (water bears) and arthropods (spiders, insects and allies), onychophorans or “velvet worms” comprise the Panarthropoda, which, together with the Cycloneuralia (nematodes, priapulids and allies), form the Ecdysozoa—the clade of molting animals ([47, 48], but see Ref. [49] for a critical review of ecdysozoan phylogeny with respect to the uncertain phylogenetic position of tardigrades and a putative paraphyly of cycloneuralians). We first screened different transcriptomic libraries of *E. rowelli* as well as the publicly available genomes of the tardigrades *Hypsibius exemplaris* and *Ramazzottius varieornatus* [50–52] for the putative NK and NKL homologs. We used the identified sequences for a phylogenetic analysis to clarify gene orthology within the entire NK family. We then generated specific probes and performed in situ hybridization on embryos of *E. rowelli* to clarify the expression patterns of NK cluster genes and the NKL gene *NK2.2* in Onychophora. Our findings provide insights into the evolutionary history and putative ancestral roles of NK cluster genes in nephrozoans.

## Results

### Identification and analysis of NK and NKL homologs in onychophorans and tardigrades

#### NK/NKL repertoire in Onychophora

**Fig. 1:**
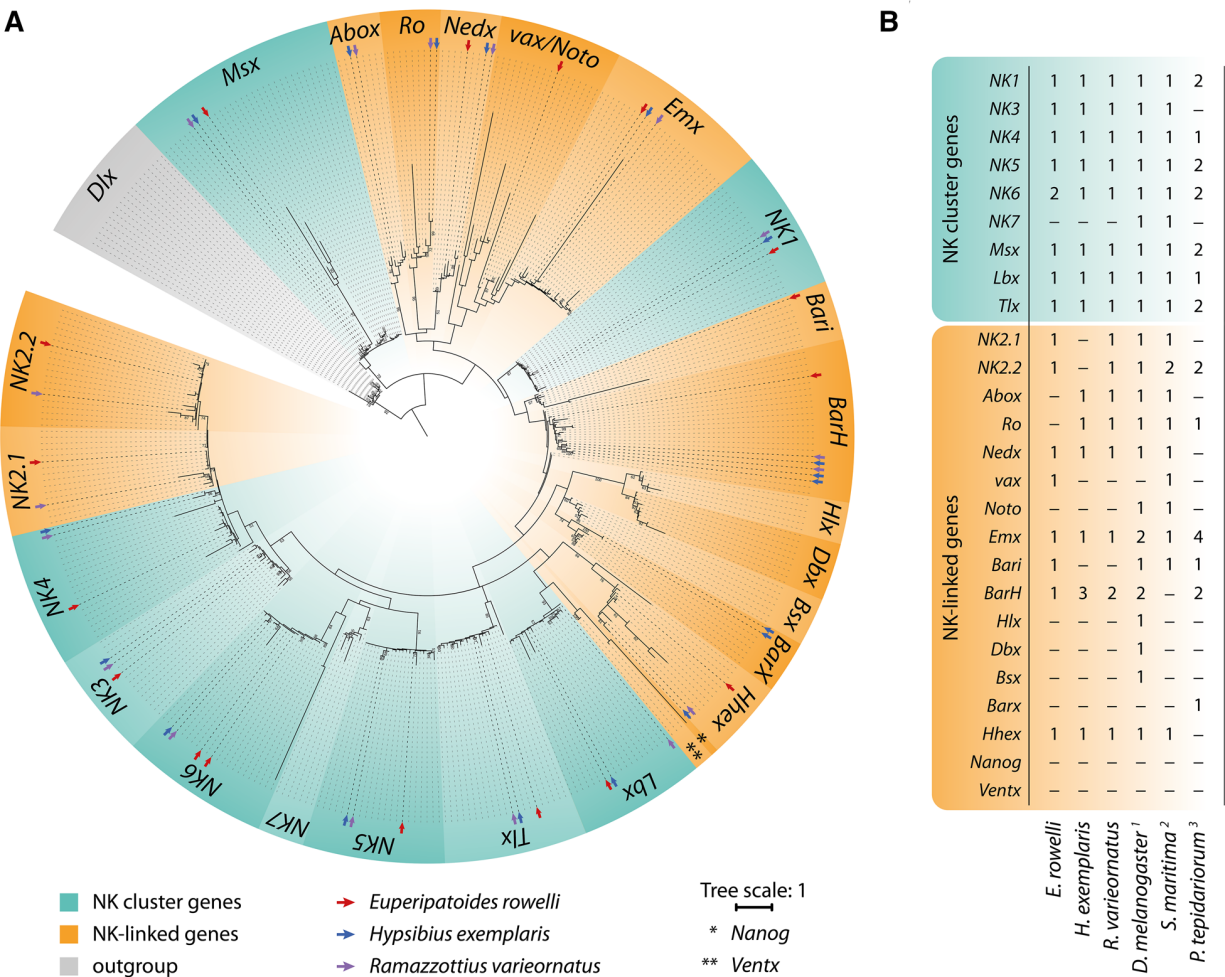
Maximum likelihood analysis of the NK cluster and NKL genes among metazoans, and summary of the NK gene repertoire in the onychophoran *E. rowelli*, and the tardigrades *H. exemplaris* and *R. varieornatus*. **A** Maximum likelihood tree of NK cluster and NKL genes to clarify the orthology of *E. rowelli*, *H. exemplaris* and *R. varieornatus* sequences (marked with red, blue and purple arrows, respectively). Bootstrap values > 50 are given at the nodes. Tree scale indicates the number of substitutions per site. **B** Summary diagram showing the NK gene complements in *E. rowelli*, *H. exemplaris*, *R. varieornatus*, *D. melanogaster*, *S. maritima* and *P. tepidariorum*. Numbers indicate the number of identified transcripts in each species; dashes indicate the absence thereof. ^1^From Ref. [131]; ^2^From Ref. [58]; ^3^From Ref. [132]. See also Additional file 1 for further information on NK gene complements in different bilaterian taxa

Screening of the transcriptome libraries of the onychophoran *E. rowelli* revealed 17 putative NK and NKL homologs (Fig. 1A, B). Reciprocal BLAST searches against the nr database of GenBank retrieved only single copies of most NK homologs, except for *NK6*, which is represented by two copies, *NK6.1* and *NK6.2*, in the transcriptome of *E. rowelli*. To clarify the orthology of the identified homologs, we conducted a phylogenetic analysis of NK and NKL genes across bilaterians (Fig. 1A). Using the ANTP-class member *distal*-*less* as an outgroup, the NK and NKL homeobox genes form a monophyletic clade. Within this clade, the individual NK and NKL genes also mostly comprise monophyletic groups with the exception of the *vax*/*Noto* assemblage, which includes the monophyletic *Emx* clade. Taken together, the NK/NKL repertoire of *E. rowelli* consists of 17 genes, including nine NK cluster genes (*NK1*, *NK3*, *NK4*, *NK5*, *Msx*, *Lbx*, *Tlx*, *NK6.1*, and *NK6.2*) and eight NKL genes (*Nedx*, *vax*, *Emx*, *Bari*, *BarH*, *Hhex*, *NK2.1*, and *NK2.2*). The NK cluster gene *NK7* and the NKL genes *Abox*, *Ro*, *Noto*, *Hlx*, *Dbx*, *Bsx*, *Barx*, *Nanog* and *Ventx* are missing from the transcriptome of *E. rowelli*.

#### NK/NKL repertoire in Tardigrada

**Fig. 2:**
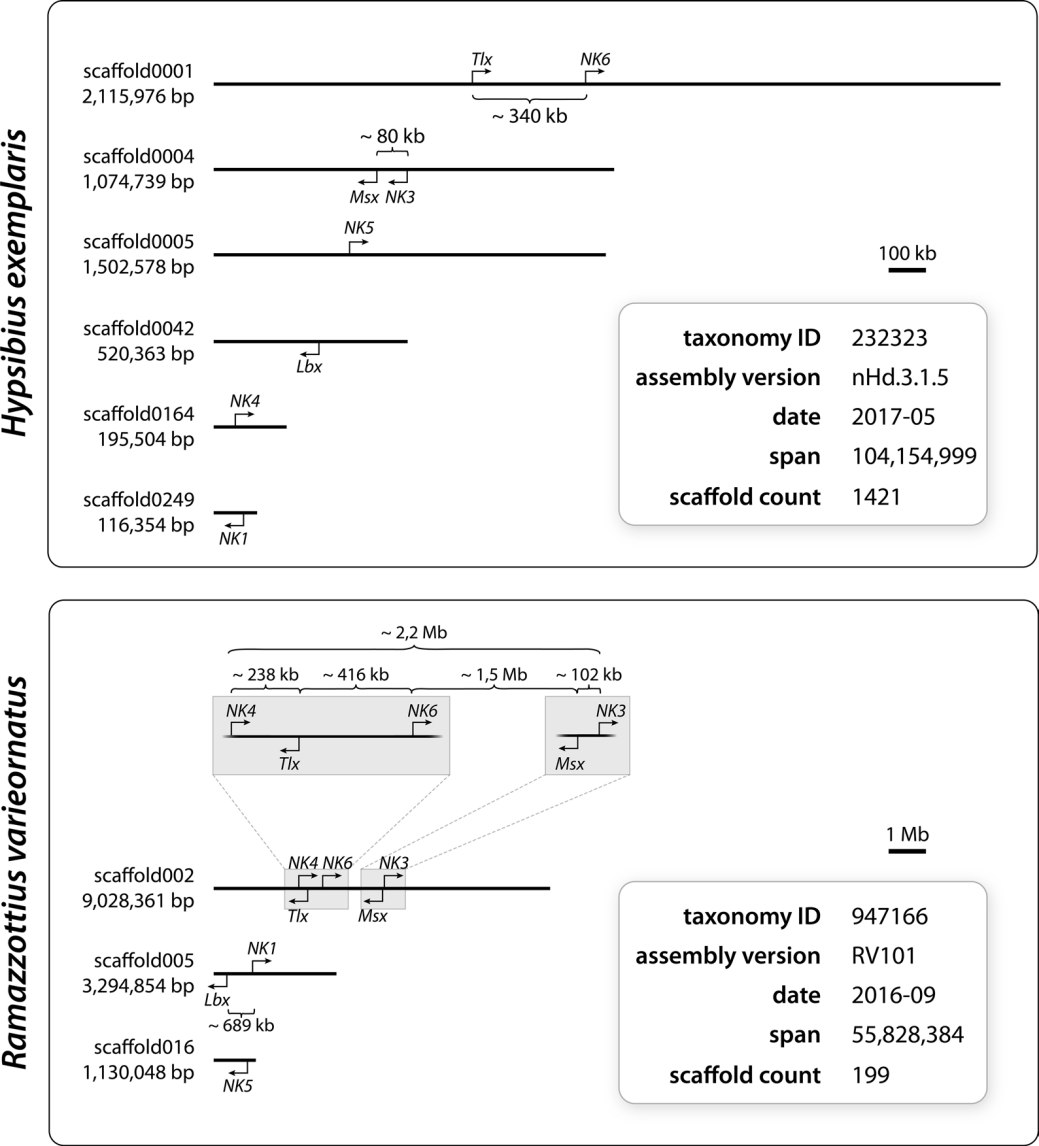
Genomic scaffolds of the tardigrades* H. exemplaris* and* R. varieornatus* showing the location and orientation of NK cluster genes. The NK genes of* H. exemplaris* are distributed on six, those of* R. varieornatus* on three different scaffolds. Numbers below the scaffold names indicate the total length of each scaffold. Numbers below or above the brackets show the distances between individual NK genes

Screening of our own transcriptomic libraries of *H. exemplaris* as well as the publicly available genomes of the two tardigrade species *H. exemplaris* and *R. varieornatus* and subsequent phylogenetic analyses revealed single copies of the eight NK cluster genes *NK1*, *NK3*, *NK4*, *NK5*, *NK6*, *Msx*, *Lbx* and *Tlx* in both species (Fig. 1A, B). In contrast to this set of NK cluster genes, which is identical in *H. exemplaris* and *R. varieornatus*, the complement of NKL genes differs between the two eutardigrade species (Fig. 1A, B). In *H. exemplaris*, we identified single copies of *Abox*, *Ro*, *Nedx*, *Emx*, *Hhex*, two copies of *Barx* and three copies of *BarH*, while *NK2.1*, *NK2.2*, *vax*, *Noto*, *Bari*, *Hlx*, *Dbx*, *Bsx*, *Nanog* and *Ventx* are missing. In *R. varieornatus*, we identified single copies of *NK2.1*, *NK2.2*, *Abox*, *Ro*, *Nedx*, *Emx*, *Hhex* and two copies of *BarH*, while *vax*, *Noto*, *Bari*, *Hlx*, *Dbx*, *Bsx*, *Nanog* and *Ventx* are missing. We further analyzed the genomic location of the identified NK cluster genes in both tardigrade species (Fig. 2; Table 1). In the genome of *H. exemplaris*, we identified two scaffolds that carry an NK gene pair each. *Tlx* and *NK6* are located on the forward strand of scaffold0001, 340,377 bp apart from each other and with a total of 59 non-NK genes between them. *Msx* and *NK3* are located on the reverse strand of scaffold0004, separated by 79,490 bp with a total of 17 non-NK genes (Fig. 2). The analysis of the *R. varieornatus* genome revealed that five NK genes, *NK4*, *Tlx*, *NK6*, *Msx* and *NK3* are located on scaffold002, spanning a region of more than 2 Mb of the scaffold with distances between the individual genes ranging from 100 kb to 1 Mb, and a total of 518 non-NK genes located in between (Table 1; Fig. 2). *NK4*, *NK6* and *NK3* are located on the forward strand, while *Tlx* and *Msx* occur on the reverse strand. Another gene pair is found on scaffold005, with *Lbx* being located on the reverse strand and *NK1* on the forward strand. Both genes are separated by ~ 700 kb and a total of 194 non-NK genes (Table 1; Fig. 2).

**Table 1:**
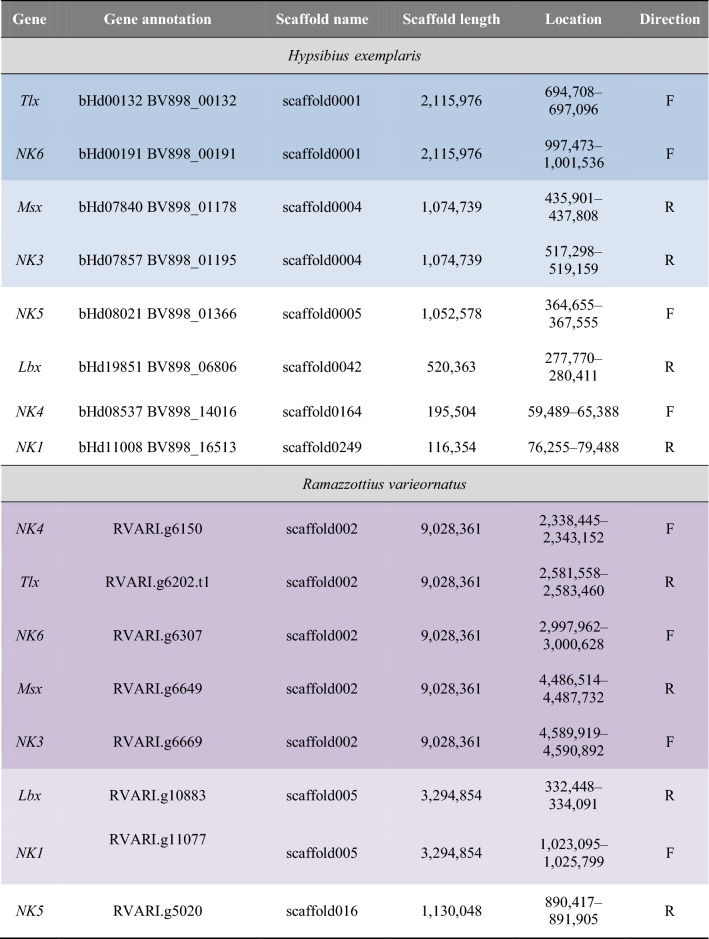
Genomic location and orientation of NK cluster genes in the tardigrades *H. exemplaris* and *R. varieornatus*. Genes that are located on the same scaffolds are highlighted in dark blue and light blue for *H. exemplaris* and dark purple and light purple for *R. varieornatus*

### Expression of NK cluster genes in embryos of the onychophoran *E. rowelli*

#### Transcription levels of NK cluster genes and the NKL gene $\text{NK2.2}$

**Fig. 3:**
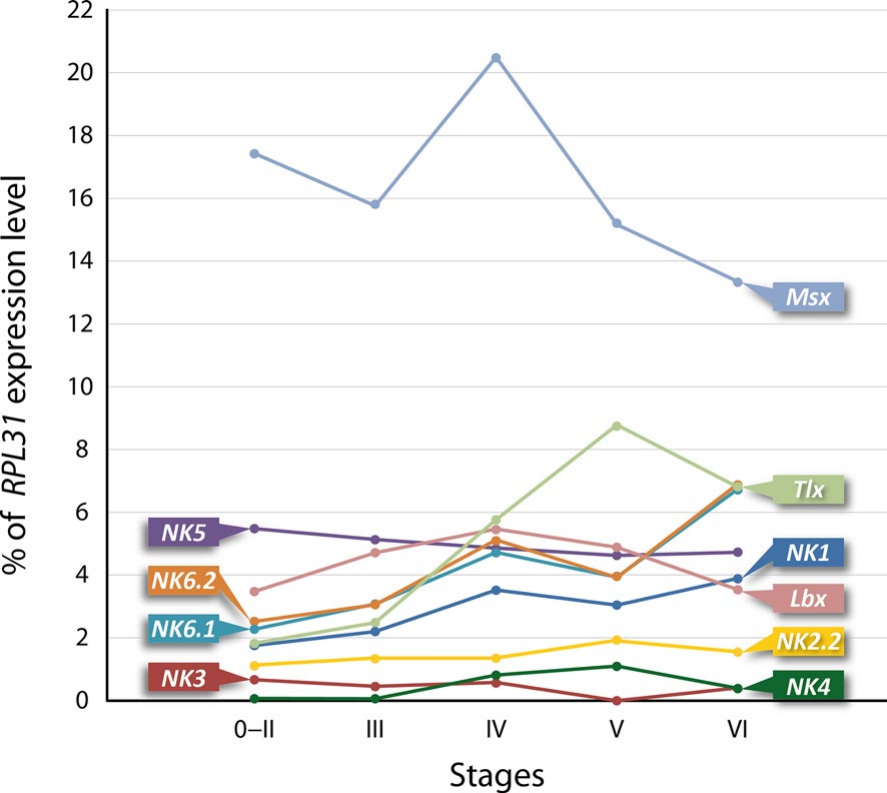
Comparative transcriptomic analysis of expression of NK cluster genes and the NKL gene *NK2.2* in the onychophoran *E. rowelli*. The diagram illustrates the relative expression levels of all identified NK cluster genes from different transcriptome libraries of partially pooled embryonic stages. The number of transcripts of *RPL31* (gene encoding a ribosomal protein) was used as a reference

To assess the abundance of the NK/NKL mRNA sequences and their transcription levels in individual developmental stages of *E. rowelli*, we performed a short read mapping using the segemehl v0.1.7 software and the ribosomal gene *RPL31* as a reference (Fig. 3; Additional file 2). *Msx* shows the highest (> 12% of the *RPL31* level) and *NK3* and *NK4* the lowest expression levels (< 1% of the *RPL31* level) throughout development, whereas *NK1*, *NK5*, *Lbx*, *Tlx, NK6.1*, *NK6.2* and *NK2.2* are expressed at levels ranging from 1 to 7%. Interestingly, the transcription levels of the two copies of *NK6* are strikingly similar throughout development (Fig. 3).

#### Expression of $\text{NK1}$

**Fig. 4:**
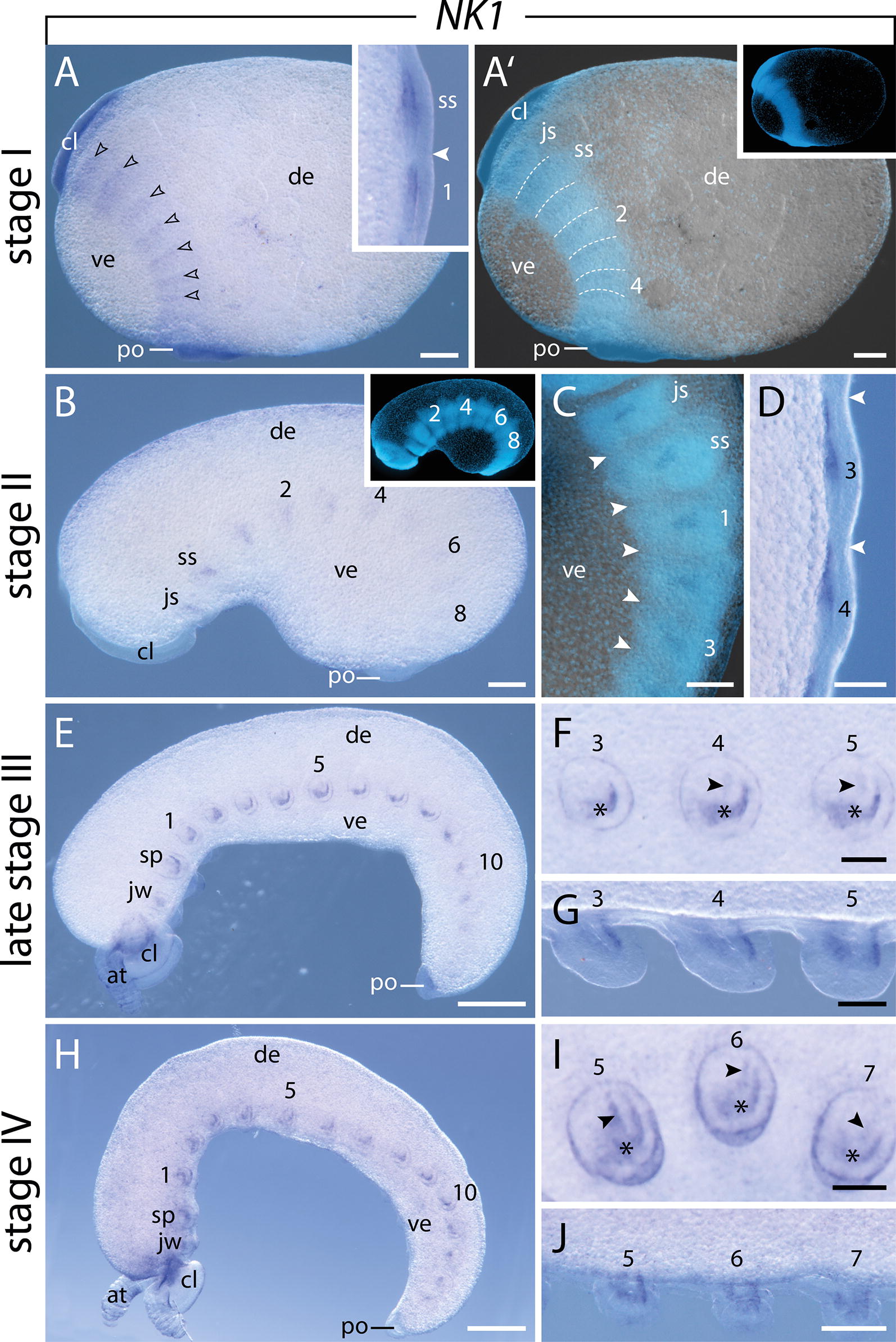
Expression of *NK1* at consecutive developmental stages in embryos of the onychophoran *E. rowelli*. Anterior is up in **A**, **A′**, **C**, **D**, and left in **B**, **E**–**J**; developing limbs are numbered. Insets in **A′** and **B** show the respective embryos stained with DAPI. **A** Stage I embryo in lateral view. Note the weak signal in the anterior segments (arrowheads). Inset shows the slime papilla segment and first leg-bearing segment in ventrolateral view. Filled arrowhead points to the transverse segmental furrow. **A′** Same embryo as in **A**, superimposed light micrograph and DAPI image. Dashed lines demarcate transverse furrows. **B** Stage II embryo in lateral view. **C** Detail of the same embryo as in **B** in lateral view. Superimposed light micrograph and DAPI image. Arrowheads indicate the position of transverse furrows. **D** Detail of stage II embryo in dorsal view. Arrowheads indicate the position of transverse furrows. **E** Late stage III embryo in lateral view. **F** Third to fifth developing legs of the same embryo as in **E** in lateral view. Note the U-shaped domain (asterisks) in all and an additional dorsal domain in the fourth and fifth legs (arrowheads). **G** Same embryo as in **F** in ventral view. **H** Stage IV embryo in lateral view. **I** Fifth to seventh developing legs of the same embryo as in **H** in lateral view. Note the U-shaped (asterisks) and an additional dorsal domain (arrowheads). **J** Same embryo as in **H** in ventral view. Abbreviations: at, developing antenna; cl, cephalic lobe; de, dorsal extra-embryonic tissue; js, jaw segment; jw, developing jaw; po, proctodeum; sp, developing slime papilla; ss, slime papilla segment; ve, ventral extra-embryonic tissue. Scale bars: **A**–**C**, **J**: 200 µm; **E**, **H**: 500 µm; **D**, **F**, **G**, **I**: 100 µm

This gene is first expressed in the mesoderm of stage I embryos in weak stripes in the anterior half of the jaw, slime papilla, and subsequent five leg-bearing segments, that are delineated by transverse segmental furrows, but not in the antennal segment (Fig. 4A, A′; note that we use the term “segmental furrows” for the morphological indentations of the body surface that are clearly seen in the embryo and that have also been referred to as “furrows,” “grooves” or “segment boundaries” in other studies [53–56], irrespective of whether or not they are homologous to the segmental/parasegmental grooves of arthropods [57]). As more segments are added posteriorly, the expression extends and the intensity of the signal increases in the anterior segments, while it is still weak in the posterior segments (Fig. 4B, C). At this stage of development, the expression is restricted to the anterior half of the mesoderm of each segment (Fig. 4C, D). As development proceeds and the limb buds start to protrude distally, a mesodermal U-shaped signal appears in each limb bud, which is larger and more prominent in the anterior limbs (Fig. 4E–H). In addition to this U-shaped domain, a weak stripe appears along the dorsal midline of the fourth and fifth legs (Fig. 4F). In later developmental stages, this expression is also visible in the limb buds posterior to the fourth and fifth leg-bearing segments but never anterior to them (Fig. 4H–J).

#### Expression of $\text{NK3}$

**Fig. 5:**
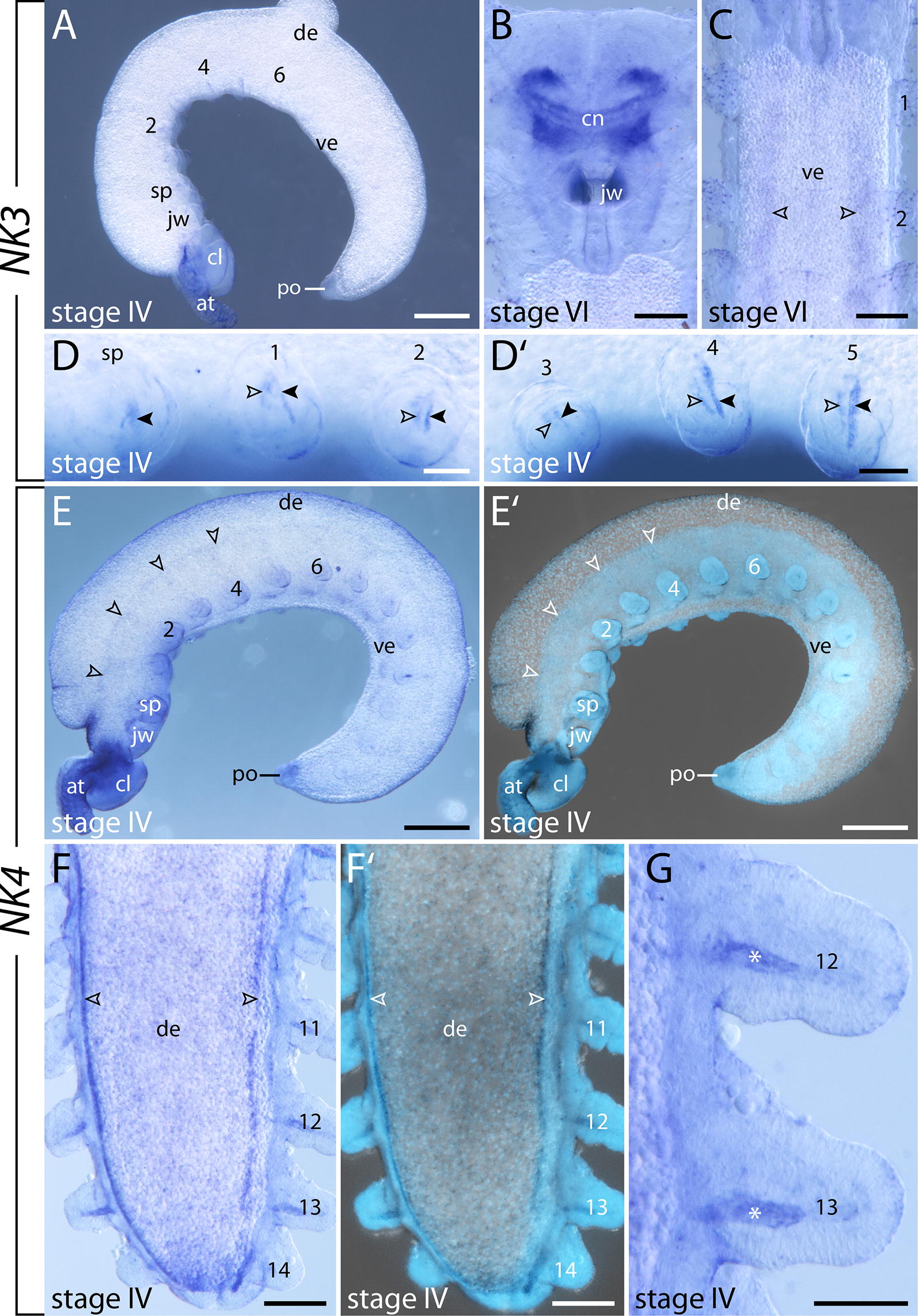
Expression of *NK3* and *NK4* at late developmental stages in embryos of the onychophoran *E. rowelli*. Anterior is left in **A**, **D**, **D′**, **E**, **E′** and up in **B**, **C**, **F**, **F′**, **G**; developing legs are numbered. **A** Stage IV embryo in lateral view. **B** Head of stage VI embryo in dorsal view. **C** Anterior region of stage VI embryo in ventral view. Arrowheads point to the expression in the anlagen of ventral nerve cords. **D** Developing slime papilla and first two legs of a stage IV embryo in lateral view. Anterior and posterior *NK3* domains are marked with empty and filled arrowheads, respectively. **D′** Third to fifth developing legs of a stage IV embryo in lateral view. Anterior and posterior *NK3* domains are marked with empty and filled arrowheads, respectively. **E** Stage IV embryo in lateral view. Empty arrowheads indicate the expression along the dorsal rims of the lateral germ bands. **E′** Superimposed light micrograph and DAPI image of the same embryo as in **E**. **F** Posterior end of a stage IV embryo in dorsal view. Arrowheads point to the expression along the dorsal rims of the lateral germ bands. **F′** Superimposed light micrograph and DAPI image of the same embryo as in **F**. **G** Detail of the 12th and 13th legs in dorsal view. Asterisks indicate the mesodermal *NK4* domains. Abbreviations: at, developing antenna; cl, cephalic lobe; cn, central brain neuropil; de, dorsal extra-embryonic tissue; jw, developing jaw; po, proctodeum; sp, developing slime papilla; ve, ventral extra-embryonic tissue. Scale bars: **A**, **E**, **E′**: 500 µm; **D**, **D′**, **G**: 100 µm; **B**, **C**, **F**, **F′**: 200 µm

*NK3* is expressed weakly throughout development, as indicated by the short read mapping (Fig. 3). Its expression is first detectable in stage IV embryos in two individual mesodermal stripes in the anterior developing limbs with one of these stripes being larger and located posteriorly and the other one being smaller and located anterior to the larger stripe (Fig. 5A, D, D′). As development proceeds, the expression in the developing limbs disappears. Additionally, a diffuse *NK3* signal appears in the developing central brain neuropil and in the anlagen of the ventral nerve cords (Fig. 5B, C).

#### Expression of $\text{NK4}$

Similar to *NK3*, the short read mapping revealed a relatively low abundance of *NK4* transcripts in the transcriptomes, indicating that its expression is weak throughout development (Fig. 3). The signal developed only after 2 days of staining and the embryos show a high level of unspecific background (Fig. 5E–G). However, at stage IV, two continuous stripes of expression appear on the dorsal side of the embryo, along the dorsal rim of the paired lateral germ bands (Fig. 5E, E′, F, F′). Stripes of expression also occur in the mesoderm of the posterior-most developing limbs (Fig. 5G). Expression of *NK4* was undetectable in other developmental stages.

#### Expression of $\text{NK5}$

**Fig. 6:**
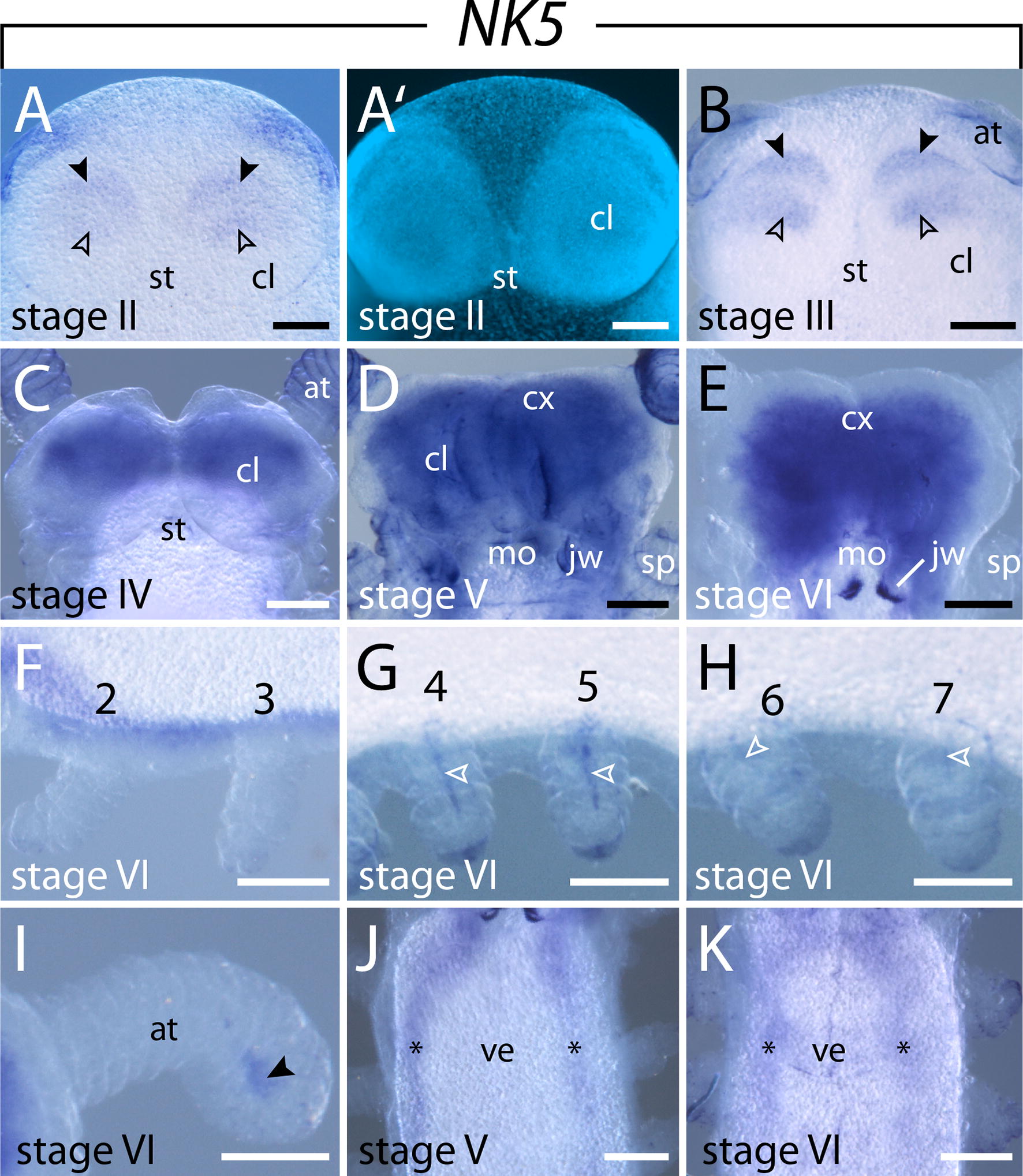
Expression of *NK5* at consecutive developmental stages in embryos of the onychophoran *E. rowelli*. Anterior is up in **A**–**E**, **I**–**K**, and left in **F**–**H**. **A, B–E** Expression in the head of subsequent developmental stages in ventral view. Empty and filled arrowheads in **A** and **B** point to two separate domains in each cephalic lobe. **A′** Same Embryo as in **A** stained with DAPI. **F–H** Second to seventh developing legs of a stage VI embryo in dorsolateral view. Arrowheads point to the mesodermal expression in the fourth to seventh legs. **I** Developing antenna of a stage VI embryo in ventral view. Note the expression in the distal mesoderm (arrowhead). **J, K** Anterior regions of stage V and VI embryos in ventral view. Note the expression in the ventral nerve cords (asterisks). Abbreviations: at, developing antenna; cl, cephalic lobe; cx, brain cortex; jw, developing jaw; mo, developing mouth; st, stomodeum; sp, developing slime papilla; ve, ventral extra-embryonic tissue. Scale bars: 200 µm

*NK5* expression is first detectable in stage II embryos in two distinct domains in the cephalic lobes that are clearly separated from each other. The anterior domain is thin and curved, while the posterior one is larger and more roundish (Fig. 6A, A′). This signal persists and intensifies in stage III embryos (Fig. 6B). As development proceeds, *NK5* expression occurs in a diffuse domain in the developing brain cortex as well as in the developing ventral nerve cords (Fig. 6C–E, J, K). Additional domains appear later in the mesoderm of the posterior developing legs from the fourth leg pair onward as well as in the distal mesoderm of the antennae (Fig. 6F–I).

#### Expression of $\text{NK6.1}$ and $\text{NK6.2}$

**Fig. 7:**
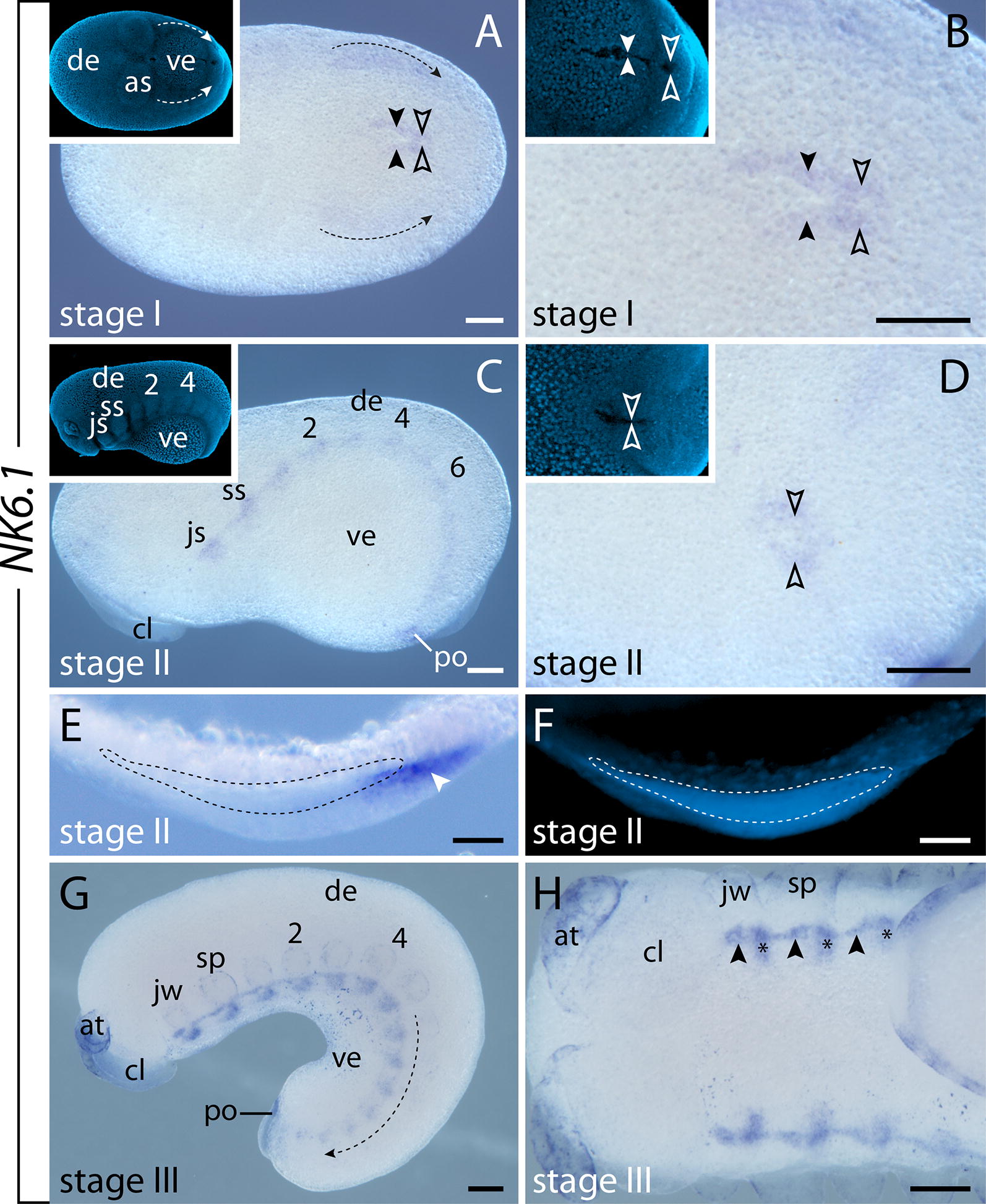
Expression of *NK6.1* at consecutive developmental stages in embryos of the onychophoran *E. rowelli*. Anterior is left in **A**–**D**, **G**, **H**; developing legs are numbered. Insets in **A**–**D** show the corresponding embryos stained with DAPI. **A** Stage I embryo in ventral view. Note the expression around the proctodeum (empty arrowheads) and the closing blastoporal slit (filled arrowheads). Arrows with dashed lines indicate decreasing signal toward the posterior. **B** Detail of proctodeum (empty arrowheads) and blastoporal slit (filled arrowheads) in the same embryo as in **A**. **C** Stage II embryo in lateral view. **D** Same embryo as in **C** in ventral view. Note the expression around the proctodeum (arrowheads). **E** Cross section of a stage II embryo, dorsal is up. Arrowhead point to the *NK6.1* expression in the ventrolateral ectoderm. Dotted line demarcates the developing nerve cord. **F** Same cross section as in E stained with DAPI. **G** Stage III embryo in lateral view. Note the decreasing signal from anterior to posterior (arrow with dashed line). **H** Same embryo as in **E** in ventral view. Small anterior and large posterior domains in the ventral ectodermal thickenings are indicated by arrowheads and asterisks, respectively. Abbreviations: as, antennal segment; at, developing antenna; cl, cephalic lobe; de, dorsal extra-embryonic tissue; js, jaw segment; jw, developing jaw; po, proctodeum; sp, developing slime papilla; ss, slime papilla segment; ve, ventral extra-embryonic tissue. Scale bars: **A**–**D**, **G**, **H**: 200 µm; **E**, **F**: 50 µm

**Fig. 8:**
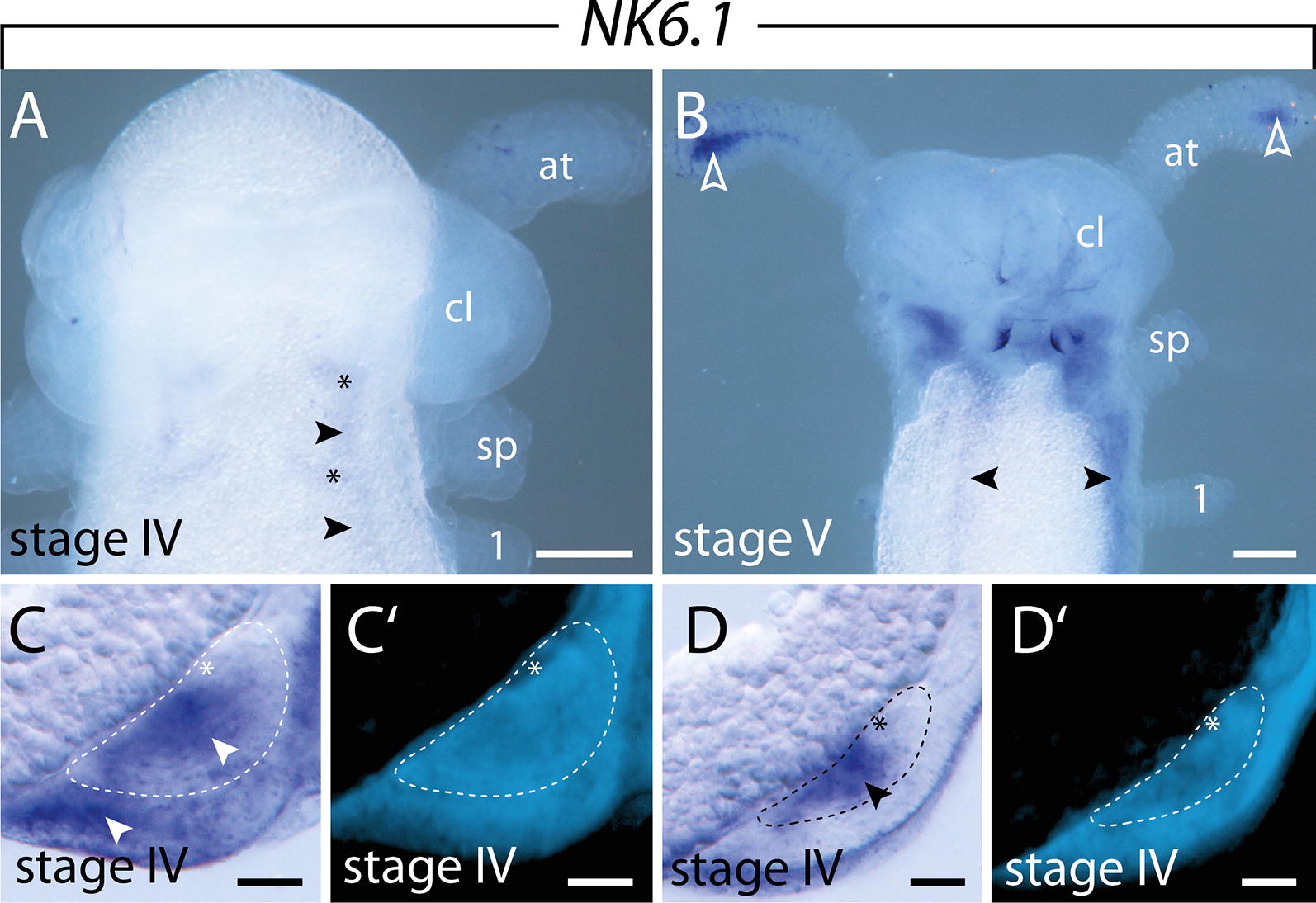
Expression of *NK6.1* at late developmental stages in embryos of the onychophoran *E. rowelli*. Anterior is up in **A** and **B**; developing legs are numbered. Developing nerve cord is indicated with dashed lines and the neuropil with asterisks in **C**–**D**′. **A** Head of stage IV embryo in ventral view. Signals in the ventral ectodermal thickenings and ventral nerve cords are indicated with asterisks and arrowheads, respectively. **B** Head of stage V embryo in ventrolateral view. Note the expression in the developing antennae (empty arrowheads) and the ventral nerve cords (filled arrowheads). **C** Cross section of a stage IV embryo showing the segmental expression in the ventral ectodermal thickenings. Arrowheads point to the expression in the ventral ectoderm and developing nerve cords. **D** Cross section of the interpedal region of a stage IV embryo. Arrowhead points to the expression in the developing nerve cords. **C**′ and **D**′ show DAPI staining of the same cross section as in **C** and **D**, respectively. Abbreviations: at, developing antenna; cl, cephalic lobe; sp, developing slime papilla. Scale bars: **A**, **B**: 200 µm; **C**–**D**′: 50 µm

The two copies of *NK6* do not only show similar transcription levels throughout development (Fig. 3), but they also exhibit the same expression patterns in all developmental stages studied (Figs. 7A–H, 8A, B; Additional file 3). *NK6.1* and *NK6.2* are initially expressed at an early stage of development, when the blastoporal slit closes. At that stage, weak expression is seen around the closing blastoporal slit (Fig. 7A, B). Additionally, a diffuse and continuous band of expression is seen along the germ bands, beginning in the jaw segment and ending in a ring-shaped domain around the proctodeum (dotted lines with arrows in Fig. 7A; Additional file 3). This expression is condensed into a thin, continuous stripe in the ventrolateral ectoderm at around the stage when the limb buds start to protrude distally (Fig. 7C–G; Additional file 3). Additionally, diffuse dot-like thickenings—a small anterior and a larger posterior one—are detected next to this band of expression in each segment, decreasing in size and intensity toward the posterior end (Fig. 7C, G; Additional file 3). As development proceeds, these diffuse dots become more defined and oval shaped in the anterior segments, while they still appear as diffuse spots in the posterior segments (Figs. 7G, H, 8A; Additional file 3). Cross sections revealed that these oval-shaped domains are located in the ventromedial ectoderm (Fig. 8C, C′), whereas the longitudinal bands of expression are confined to the developing nerve cords (Fig. 8D, D′). In later developmental stages, the expression of *NK6.1* and *NK6.2* persists in each ventral nerve cord and expands anteriorly into the jaw segment, while the oval-shaped ectodermal domains disappear (Fig. 8A, B; Additional file 3). An additional domain appears in the distal mesoderm of the developing antennae (Fig. 8B).

#### Expression of $\text{Msx}$

**Fig. 9:**
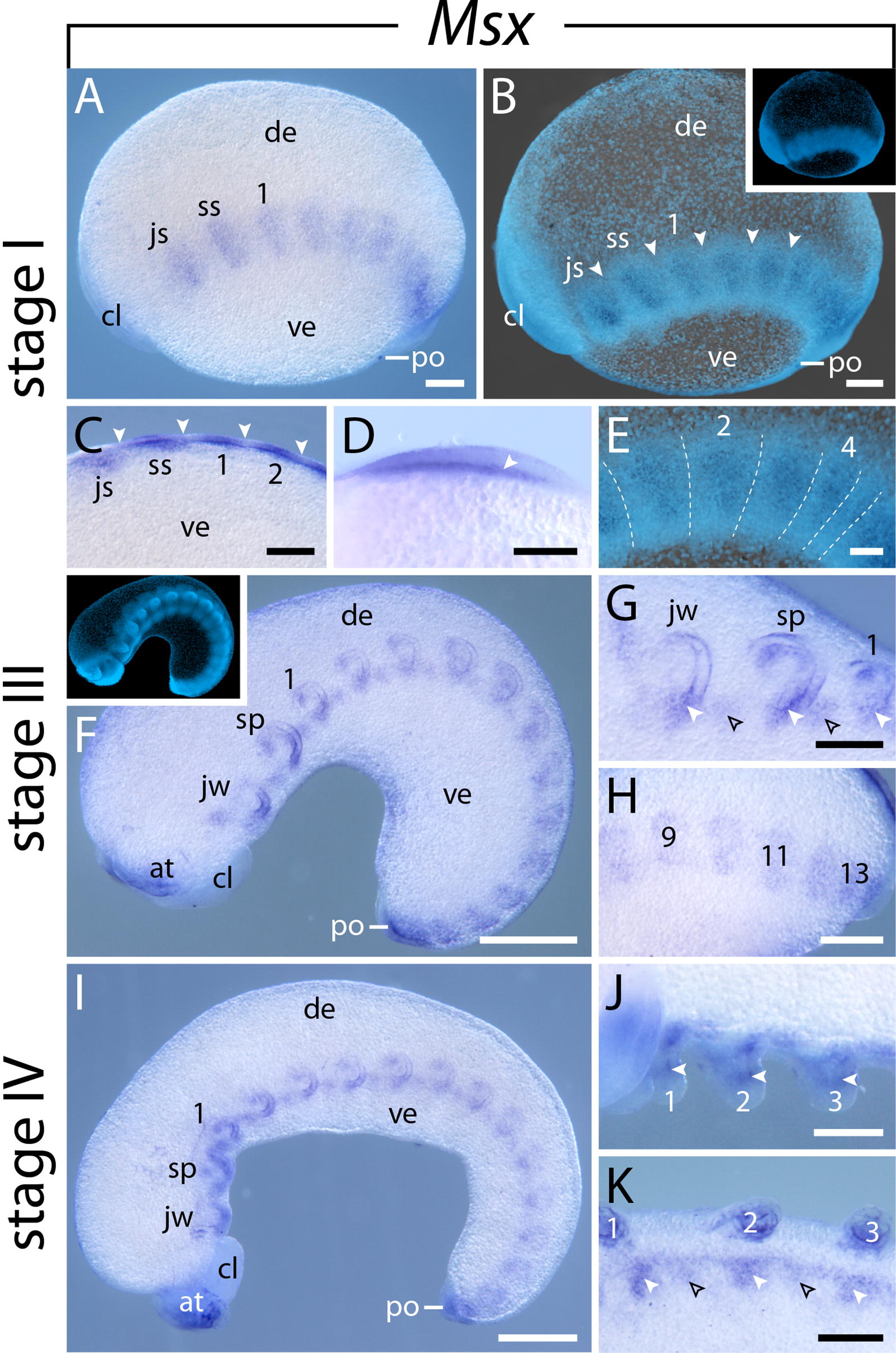
Expression of *Msx* at consecutive developmental stages in embryos of the onychophoran *E. rowelli*. Anterior is left in **A**–**C**, **E**–**K**; developing legs are numbered. Insets in **B** and **F** show the respective embryos stained with DAPI. **A** Stage I embryo in lateral view. **B** Same embryo as in **A** in ventrolateral view, superimposed light micrograph and DAPI image. Arrowheads point to the transverse furrows. **C** Detail of a stage I embryo in ventral view. Arrowheads point to transverse furrows. **D** Cross section of the first leg-bearing segment, dorsal is left. Arrowhead points to the expression in the mesoderm. **E** Detail of a stage I embryo in lateral view. Superimposed light micrograph and DAPI image. Transverse furrows are indicated by dashed lines. **F** Stage III embryo in lateral view. **G** Detail of the same embryo as in **F** in ventrolateral view. Anterior and posterior domains in the ventral ectodermal thickenings are marked by empty and filled arrowheads, respectively. **H** Posterior end of stage III embryo in lateral view. **I** Stage IV embryo in lateral view. **J** Detail of the same embryo as in **I** in ventral view. Arrowheads point to mesodermal domains in the limb buds. **K** Stage IV embryo in ventrolateral view. Anterior and posterior domains in the ventral ectodermal thickenings are marked with empty and filled arrowheads, respectively. Abbreviations: at, developing antenna; cl, cephalic lobe; de, dorsal extra-embryonic tissue; js, jaw segment; jw, developing jaw; po, proctodeum; sp, developing slime papilla; ss, slime papilla segment; ve, ventral extra-embryonic tissue. Scale bars: **A**–**C**, **G**, **H**, **J**, **K**: 200 µm; **F**, **I**: 500 µm; **D**, **E**: 100 µm

**Fig. 10:**
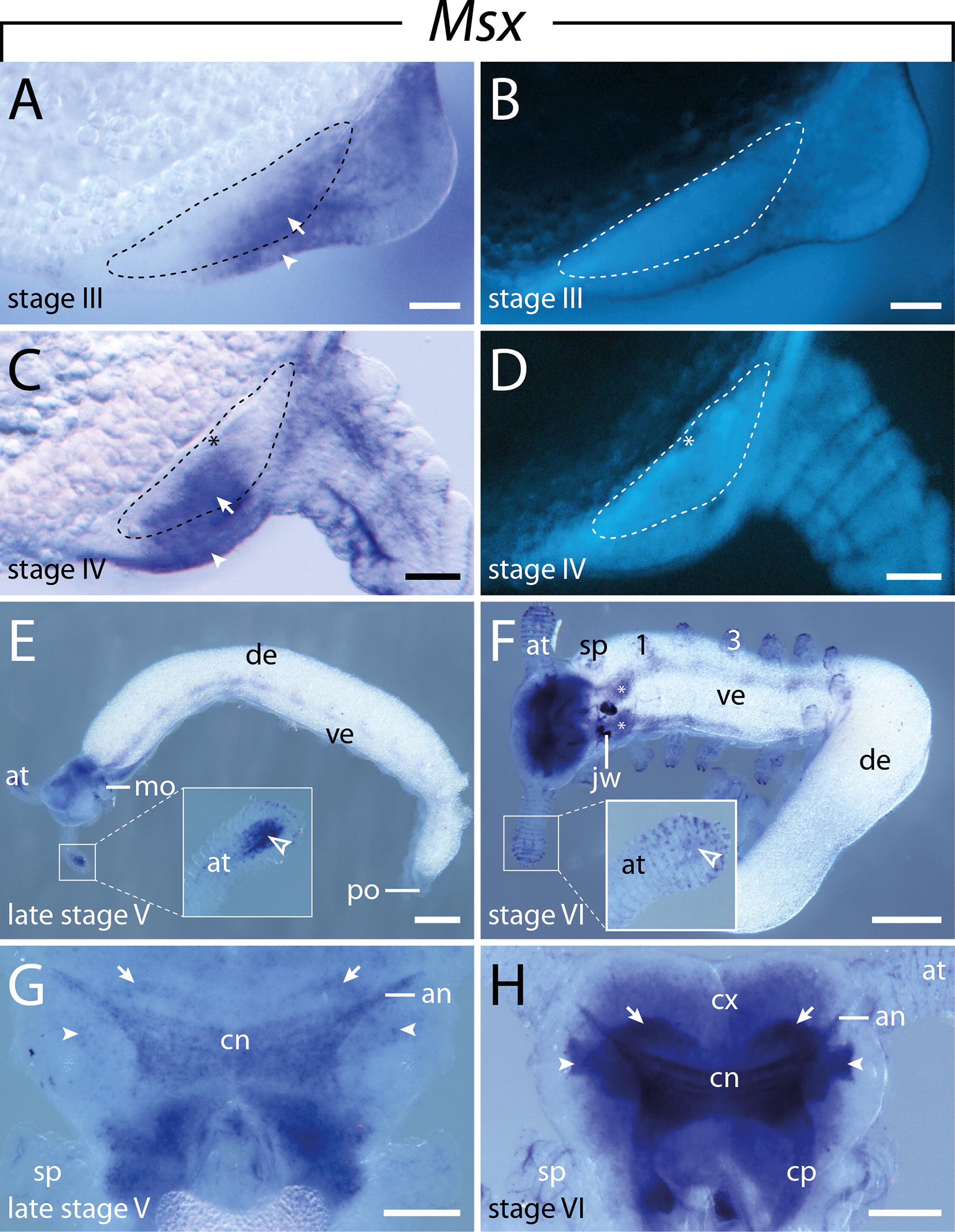
Expression of *Msx* at consecutive developmental stages in embryos of the onychophoran *E. rowelli*. Anterior is left in **E**, **F**, and up in **G**, **H**; developing legs are numbered. **A** Cross section of a stage III embryo showing the expression in the ventrolateral ectoderm (arrowhead) and developing nerve cord (arrow). **B** Same cross section as in **A** stained with DAPI. Developing nerve cord is indicated with dashed lines. **C** Cross section of a stage IV embryo showing the expression in the ventral ectoderm (arrowhead) and developing nerve cord (arrow). **D** Same cross section as in **C** stained with DAPI. Developing nerve cord is indicated with dashed lines, developing neuropil is indicated with asterisks in **A** and **B**. **E** Late stage V embryo in ventrolateral view. Note the strong expression in the distal portion of the developing antennae (inset). **F** Stage VI embryo in ventrolateral view. Note that the signal is stronger in the slime papilla segment (asterisks) than in the subsequent segments. Note also the decreased expression in the developing antennae (inset) as compared to the embryo in **E**. **G** Head of late stage V embryo in dorsal view. **H** Head of a stage VI embryo in dorsal view. Arrows in **G** and **H** point to uncharacterized anterior brain neuropils; arrowheads indicate the putative olfactory lobes. Abbreviations: an, antennal tracts; at, developing antenna; cn, central brain neuropil; cp, connecting piece (cf. Ref. [141]; “medullary connective” sensu Whitington PM and Mayer G [111]); cx, brain cortex; de, dorsal extra-embryonic tissue; jw, developing jaw; mo, mouth; po, proctodeum; sp, developing slime papilla; ve, ventral extra-embryonic tissue. Scale bars: **A**–**D**: 50 µm; **E**, **F**: 500 µm; **G**, **H**: 200 µm

This gene is initially expressed in stage I embryos in each segment except for the antennal segment (Fig. 9A, B). While the expression pattern is still diffuse and continuous in the posterior-most part of the lateral germ bands, i.e., it is not subdivided into segmental domains, the domains are clearly separate from each other in the anterior-most segments, where they occupy the posterior two-thirds of each segment (Fig. 9A–E). In stage III embryos, expression occurs in the mesoderm of the developing limbs, including the antennae (Fig. 9F–H). Additionally, similar to *NK6.1* and *NK6.2*, paired dot-like *Msx* domains—a small anterior and a larger posterior one—appear in the ventrolateral ectoderm (Figs. 9F, G, 10A, B). As the limb buds grow distally, the expression in the developing limbs becomes restricted to the proximal portion and is absent from the distal portion of each limb (Fig. 9I, J). Furthermore, a continuous band of expression appears in the nerve cords lateral to the paired dot-like domains. Cross sections revealed that the paired dot-like domains are restricted to the center of the ventral ectoderm, while the longitudinal bands of expression are located in the developing nerve cords (Figs. 9K, 10A, B). In later developmental stages, *Msx* expression disappears from the developing limbs apart from a distinct domain in the distal mesoderm of the developing antennae (Fig. 10C, D). At this stage of development, *Msx* expression is initiated in the developing brain, including the central brain neuropil, the antennal tracts, the brain cortex, and the connecting pieces that link the brain to the ventral nerve cords (Fig. 10E–H). Furthermore, *Msx* expression persists in the ventral nerve cords with a decreasing intensity from anterior to posterior, while the paired dot-like domains in the ventral ectoderm have disappeared (Fig. 10C). Later in development, the intensity of expression in the developing brain and ventral nerve cords increases, while the signal in the distal mesoderm of the developing antennae disappears (Fig. 10D, F).

#### Expression of $\text{Lbx}$

**Fig. 11:**
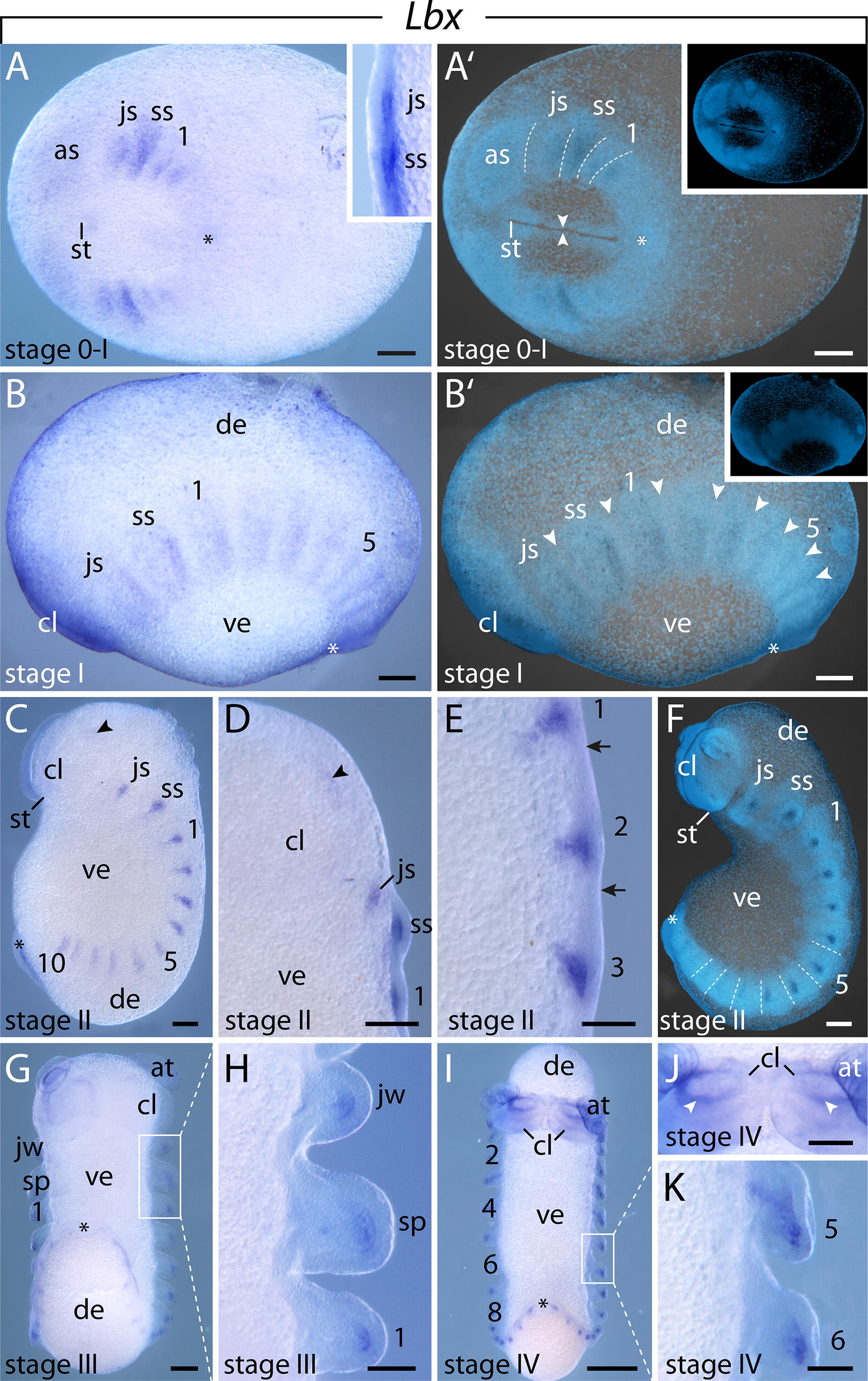
Expression of *Lbx* at consecutive developmental stages in embryos of the onychophoran *E. rowelli*. Anterior is left in **A**–**B′**, and up in **C**–**K**; developing legs are numbered. Asterisks point to the proctodeum. Insets in **A′** and **B′** show the respective embryo stained with DAPI. **A** Stage 0–I embryo in ventral view. Inset shows the jaw and slime papilla segments in ventrolateral view. **A′** Same embryo as in **A**, superimposed light micrograph and DAPI image. Dashed lines indicate transverse furrows. **B** Stage I embryo in lateral view. **B′** Same embryo as in **B**, superimposed light micrograph and DAPI image. Arrowheads point to transverse furrows. **C** Stage II embryo in lateral view. Note the weak signal in the developing antenna (arrowhead). **D** Head of stage II embryo in ventral view. Note the weak signal in the developing antenna (arrowhead). **E** Detail of stage II embryo in ventrolateral view. Arrows indicate transverse furrows. **F** Stage II embryo in lateral view, superimposed light micrograph and DAPI image. Dashed lines demarcate transverse furrows in the posterior segments. **G** Stage III embryo in ventral view. **H** Anterior segments of the same embryo as in **G** in ventral view. **I** Stage IV embryo in ventral view. **J** Head of a stage IV embryo in ventral view. Arrowheads point to the expression in the cephalic lobes. **K** Fifth and sixth developing legs of stage IV embryo in ventral view. Abbreviations: as, antennal segment; at, developing antenna; cl, cephalic lobe; de, dorsal extra-embryonic tissue; js, jaw segment; jw, developing jaw; sp, developing slime papilla; ss, slime papilla segment; st, stomodeum; ve, ventral extra-embryonic tissue. Scale bars: **A**–**G**, **J**: 200 µm; **H**, **K**: 100 µm; **I**: 500 µm

Initially, *Lbx* is expressed in the posterior half of the mesoderm of the jaw segment, slime papilla segment and the first two leg-bearing segments (Fig. 11A, A′). As more segments are added, the expression occurs in all segments that are delineated by transverse furrows (Fig. 11B–F). These domains are restricted to the posterior mesoderm of each segment (Fig. 11B′, E, F). Additionally, a weak signal is detected in the anlagen of the antennae (Fig. 11C, D). Later in development, when the limb buds start to grow distally, the expression in the limb buds becomes restricted to the distal mesoderm (Fig. 11G, H). At around stage IV, these domains expand toward the proximal portion of the anterior limbs, while they are still restricted only to the distal portion of the posterior limbs (Fig. 11I, K). Additionally, a ring-like expression appears in the anterior portion of the cephalic lobes at this stage of development (Fig. 11J).

#### Expression of $\text{Tlx}$

**Fig. 12:**
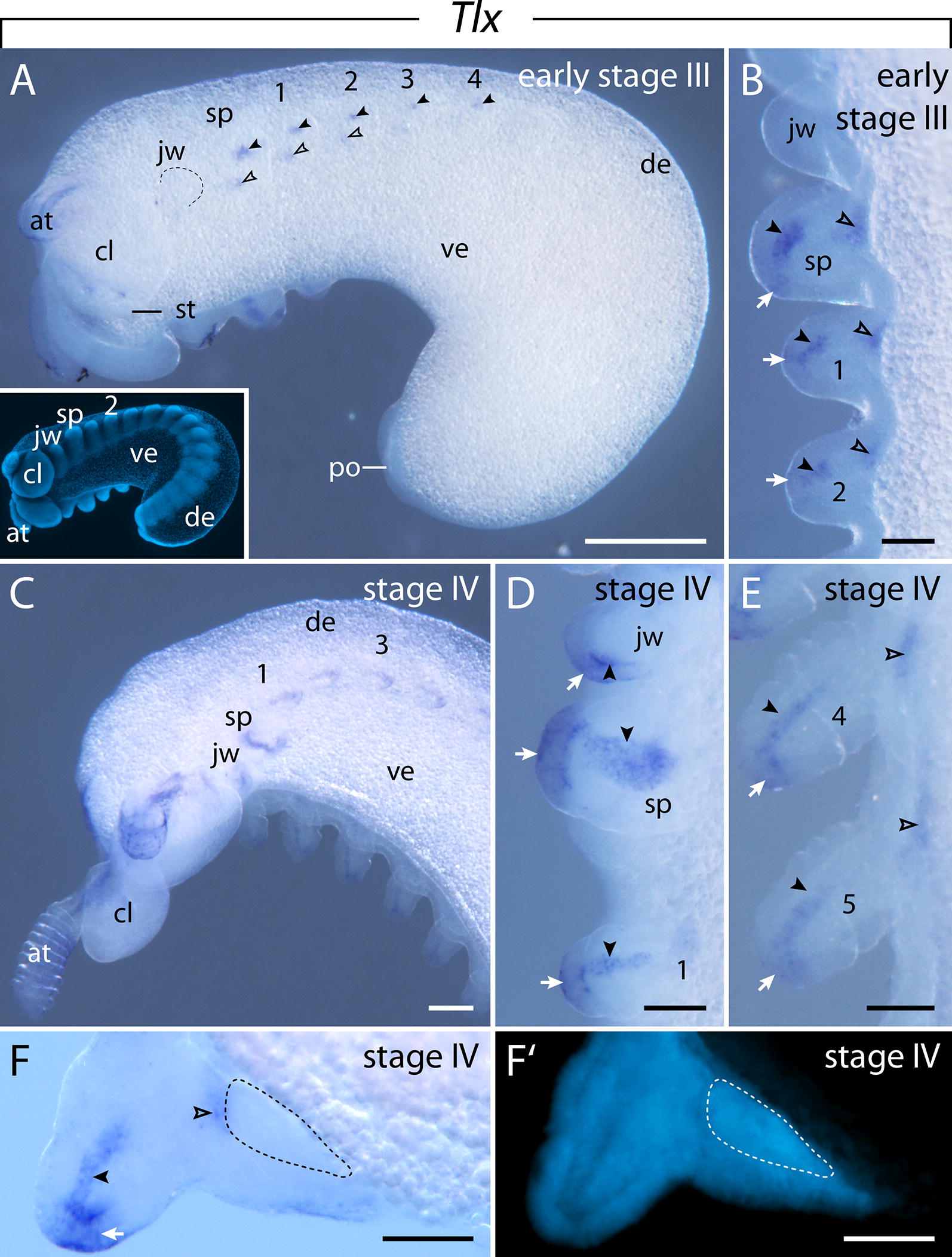
Expression of *Tlx* at consecutive developmental stages in embryos of the onychophoran *E. rowelli*. Anterior is left in **A**, **C**, and up in **B**, **D**, **E**; developing legs are numbered. **A** Stage III embryo in ventrolateral view. Note the two individual domains in the anterior limb buds (empty and filled arrowheads). Inset shows the same embryo stained with DAPI. **B** Detail of anterior limb buds of stage III embryo in ventral view. Note the ectodermal (arrows) and mesodermal domains (filled arrowheads) in the tips, and a separate proximal domain (empty arrowheads) in each limb bud. **C** Anterior end of a stage IV embryo in ventrolateral view. **D** Detail of the anterior limb buds of a stage IV embryo. Ventrolateral view. Note the ectodermal (arrows) and elongated mesodermal domains (arrowheads). **E** Developing fourth and fifth legs of a stage IV embryo in ventrolateral view. Note the ectodermal (arrows) and mesodermal domains (filled arrowheads) in the tips, and a separate proximal domain (empty arrowheads) in each limb bud. **F** Cross section of the developing fifth leg, dorsal is up. Note the ectodermal (arrow) and mesodermal domains (filled arrowhead) in the tips, and a separate proximal domain (empty arrowhead). **F**′ Same cross section as in **F** stained with DAPI. Developing nerve cord is indicated with dashed lines in **F** and **F**′. Abbreviations: at, developing antenna; cl, cephalic lobe; de, dorsal extra-embryonic tissue; jw, developing jaw; po, proctodeum; sp, developing slime papilla; st, stomodeum; ve, ventral extra-embryonic tissue. Scale bars: **A**: 500 µm; **B**, **D**, **E**, **F**, **F**′: 100 µm; **C**: 200 µm

The initial *Tlx* expression appears as two domains in the anterior mesoderm and one domain in the distal ectoderm of the anterior limb anlagen of the early stage III embryo (Fig. 12A, B). The two mesodermal domains show a stronger signal than the ectodermal domain at stage III (Fig. 12B). However, the signal in the ectoderm increases later in development and reaches a similar level to that in the mesoderm in stage IV and V embryos (Fig. 12D, E). As development proceeds, additional limb buds begin to express *Tlx* in two mesodermal domains and one distal ectodermal domain (Fig. 12C, F, F′). At this stage of development, the distal mesodermal domain of the anterior limb buds extends proximally, while the corresponding domains in the posterior limbs are still restricted to the tip (Fig. 12D, E, F, F′). The ectodermal expression in the jaw anlagen is delayed with respect to the subsequent segments, as it is first detectable at stage IV (Fig. 12C, D). Moreover, the corresponding domain occurs posterior rather than distally in the anlagen of the jaws (Fig. 12D).

#### Expression of $\text{NK2.2}$

**Fig. 13:**
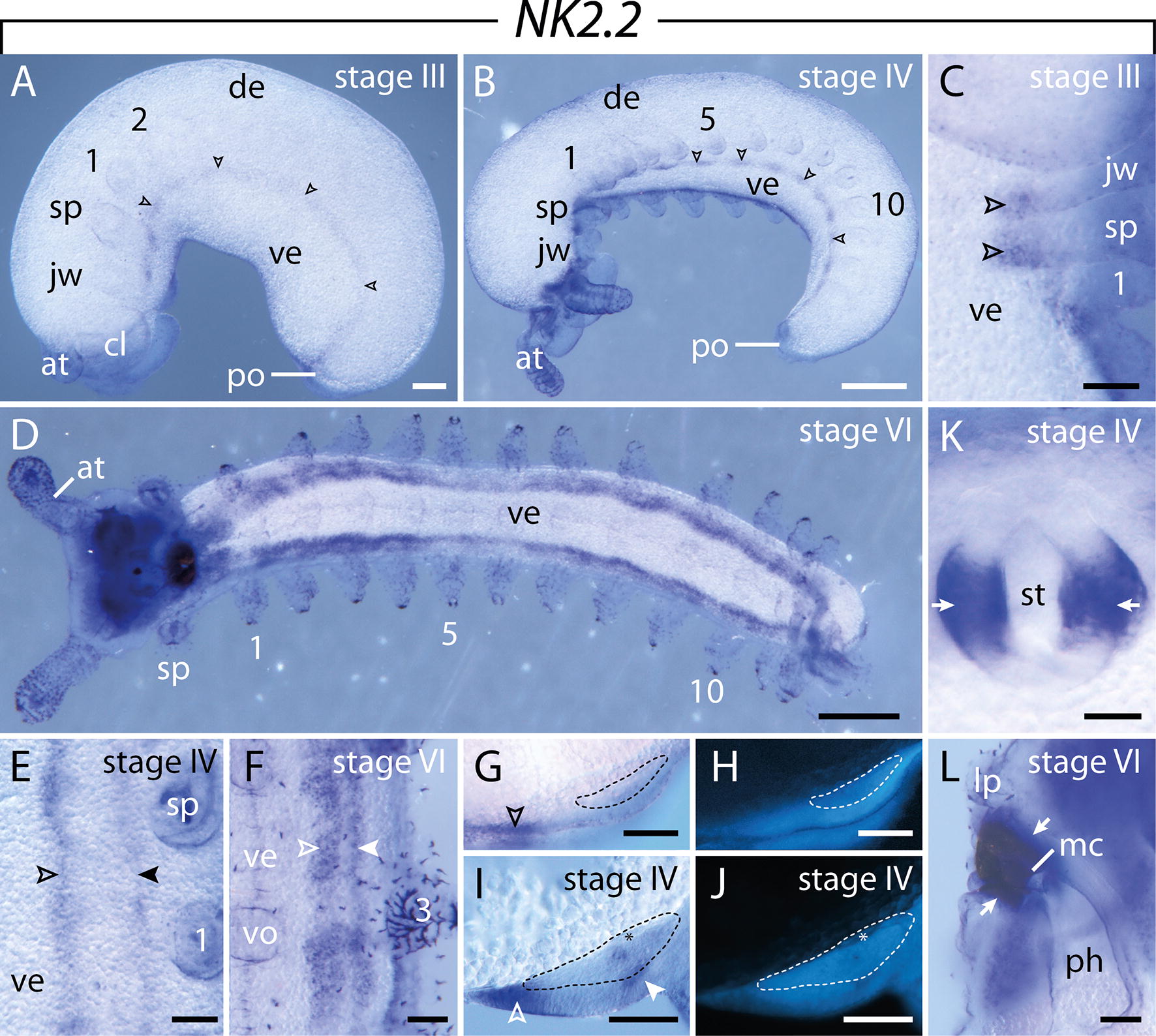
Expression of *NK2.2* at consecutive developmental stages in embryos of the onychophoran *E. rowelli*. Anterior is left in **A**, **B**, **D** and up in **C**, **E**, **F**, **K**, **L**; developing legs are numbered. Empty and filled arrowheads point to the medial and lateral domain in the ventral nerve cords, respectively. **A** Stage III embryo in lateral view. **B** Stage IV embryo in ventrolateral view. **C** Stage III embryo in ventral view. **D** Stage VI embryo in ventral view. **E** Stage IV embryo in ventral view. **F** Stage VI embryo in ventral view. **G** Cross section of a stage III embryo, dorsal is up. **H** Same cross section as in **G** stained with DAPI. **I** Cross section of a stage IV embryo, dorsal is up. **J** Same cross section as in **I** stained with DAPI. Dashed lines indicate developing nerve cords in **G**–**J**. **K** Stage IV embryo in ventral view, detail of the stomodeum. Arrows point to the domains on either side of the stomodeum. **L** Sagittal section of the head of a stage VI embryo, dorsal is right. Note the expression in the mouth cavity (arrows). Abbreviations: at, developing antenna; cl, cephalic lobe; de, dorsal extra-embryonic tissue; jw, developing jaw; lp, developing lip; mc, mouth cavity; ph, developing pharynx; po, proctodeum; sp, developing slime papilla; st, stomodeum; ve, ventral extra-embryonic tissue; vo, developing ventral organ. Scale bars: **A**: 200 µm; **B**, **D**: 500 µm; **C**: 50 µm; **E**–**L**: 100 µm

The expression of *NK2.2* is initiated in the developing ventral nerve cords of stage II embryos, beginning in the jaw segment. At this stage, a single continuous stripe of expression is seen in most segments, with additional thickenings in the jaw and slime papilla segments (Fig. 13A, C). As development proceeds, this stripe of *NK2.2* expression is subdivided into a stronger medial and a weaker lateral domain (Fig. 13B, D–F). Cross-sectioned embryos show that the medial domain is restricted to the medial ectoderm in early and late developmental stages, whereas the lateral domain corresponds to the expression pattern in the lateral ectoderm as well as in the mediolateral portion of the nerve cord in later developmental stages (Fig. 13G–J). This paired pattern persists until late in development (Fig. 13D, F). Additionally, a bilateral pattern of expression is seen in the walls of the stomodeum at stage IV (Fig. 13K), whereas the expression occurs in the walls of the entire mouth cavity at stage VI (Fig. 13L).

## Discussion

### NK and NKL gene repertoires in Onychophora and Tardigrada

Our searches and phylogenetic analyses revealed a total of 17 NK and NKL genes in the onychophoran *E. rowelli* and the tardigrade *R. varieornatus*, whereas the other analyzed tardigrade species, *H. exemplaris*, possesses 18 genes. We identified nine NK cluster genes, including single copies of *NK1*, *NK3*, *NK4*, *NK5*, *Msx*, *Lbx*, *Tlx* and two different transcripts of *NK6*, in the transcriptome of *E. rowelli*. The genomes of the two tardigrade species and the transcriptome of *H. exemplaris* show identical sets of eight NK cluster genes, which comprise single copies of *NK1*, *NK3*, *NK4*, *NK5*, *NK6*, *Msx*, *Lbx* and *Tlx*. These results show that, apart from the duplication of *NK6* in onychophorans (or the onychophoran subgroup containing *E. rowelli*), onychophorans and tardigrades possess an identical set of eight NK cluster genes, whereas *NK7* is missing in both taxa. However, the presence of *NK7* in the genomes of several arthropods [Additional file 1; 23, 58] as well as other bilaterians [Additional file 1; 24, 59] suggests that a complete “protoNK cluster” [7, 23, 60, 61] consisting of nine genes was present in the last common ancestor of Panarthropoda and that *NK7* was most likely lost in the onychophoran and tardigrade lineages.

Interestingly, localization of NK cluster genes in the genomes of the two tardigrade species revealed that most of these genes are located on different scaffolds. While the NK cluster genes are encoded on six scaffolds in *H. exemplaris* (with four scaffolds containing one and two scaffolds including two NK genes each), they are encoded on three scaffolds in *R. varieornatus* (with each scaffold containing one, two and five NK genes, respectively). The gene pairs *NK3/NK4* and *NK6/Tlx* occur in both tardigrade species, suggesting that these pairs were present in the genome of the last common ancestor of the two species. However, even those NK genes that are located on the same scaffold do not neighbor each other but lie at a distance ranging from several kb to up to ~ 1.5 Mb. Moreover, large numbers of non-NK genes are interspersed between the individual NK genes. For comparison, the NK cluster of the fruit fly *D. melanogaster* spans only about 180 kb and contains only four non-NK genes [18, 23]. We therefore conclude that the NK cluster might have been fragmented in tardigrades. Similar breakups have been reported for the tardigrade Hox cluster (see supplementary figure S3 in Ref. [62]) as well as for the Hox and ParaHox clusters in other bilaterians, including the fruit fly *D. melanogaster*, the nematode *C. elegans* and the tunicate *Ciona intestinalis* [63–65]. Comparative analyses of the genomes of *H. exemplaris* and *R. varieornatus* generally revealed a low level of synteny, which might be due to extensive intrachromosomal rearrangements similar to those in *C. elegans* [66]. It has been hypothesized that such breakups of gene clusters are correlated with rapid modes of embryogenesis, possibly due to the loss of temporal collinearity and corresponding regulatory mechanisms in these taxa [63]. Since tardigrades also show rapid embryonic development (4–5 days in *H. exemplaris* [67]), this might be indeed due to the disintegrated homeobox clusters in these animals.

In contrast to the two tardigrade species, well-assembled genomic data are currently unavailable for Onychophora. Since neither the location nor the orientation of NK genes are known in *E. rowelli*, it remains unclear whether or not the ancestral NK cluster has been retained in the onychophoran genome.

In contrast to the almost identical sets of NK cluster genes in onychophorans and tardigrades, the NKL gene complement is variable in the three species studied. While *E. rowelli* shows eight NKL genes (*NK2.1*, *NK2.2, Nedx*, *vax*, *Emx*, *Bari*, *BarH* and *Hhex*), we found ten NKL genes in *H. exemplaris* (*Abox*, *Ro*, *Nedx*, *Emx*, *Hhex*, plus two copies of *Barx* and three copies of *Barh*) and nine in *R. varieornatus* (*NK2.1*, *NK2.2*, *Abox*, *Ro*, *Nedx*, *Emx*, *Hhex*, plus two copies of *Barh*). These results, together with the NKL complements of various arthropods, indicate that the last common ancestor of Panarthropoda might have possessed single copies of each NKL gene except for *Nanog* and *Ventx*, which were missing, and that several gene losses and gene duplication events occurred in different lineages (Fig. 1B; Additional file 1). The high variation of the NKL gene repertoire in tardigrades, onychophorans and arthropods suggests that the NKL genes are more prone to evolutionary changes than the NK cluster genes.

### Segment polarity-like expression patterns of *NK1*, *Lbx* and *Msx* in the onychophoran *E. rowelli*

**Fig. 14:**
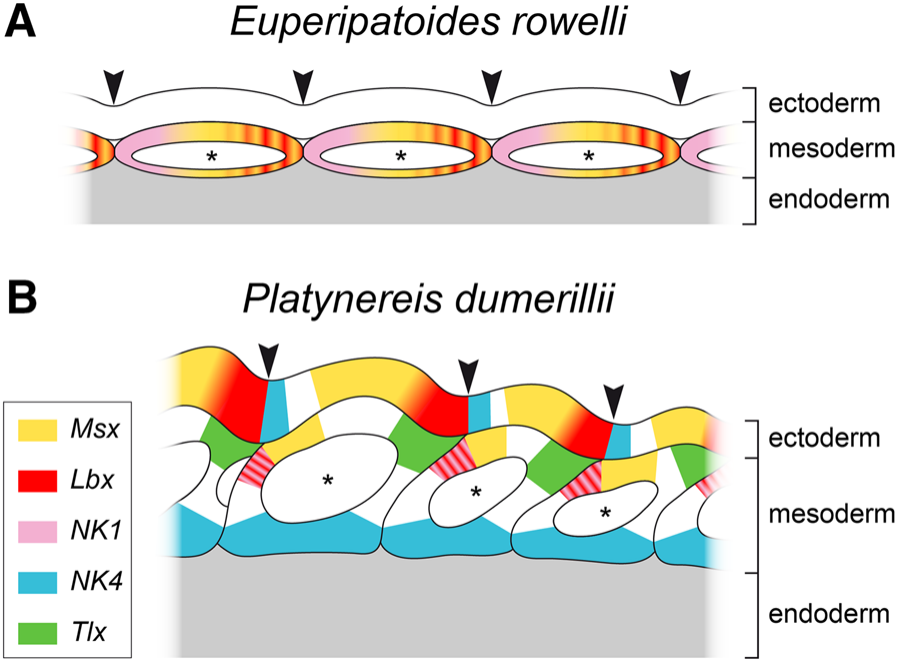
Comparison of the segment polarity patterns of expression of *Msx*, *Lbx*, *NK1*, *NK4* and *Tlx* in the onychophoran *E. rowelli* and the annelid *Platynereis dumerilii*. Diagram of *P. dumerilii* modified from Ref. [19]. The cavities of somites (= coelomic cavities) are labeled with asterisks. Note that the somites are shifted anteriorly in *P. dumerilii*, while they align with transverse furrows (arrowheads) in *E. rowelli*. **A**, **B** Note that the expression of *Msx*, *Lbx* and *NK1* is restricted to the mesoderm in *E. rowelli*, whereas *Msx*, *Lbx* and *NK4* show additional ectodermal segment polarity domains in *P. dumerilii*

Our data revealed that *NK1*, *Lbx* and *Msx* are expressed early in development and show a stereotypic pattern in the mesoderm (somites) of the onychophoran embryo. Each somite of *E. rowelli* exhibits a wide anterior *NK1* domain, followed by a diffuse medial *Msx* domain and a posterior *Lbx* domain (Fig. 14A). This pattern strongly resembles the expression of the so-called segment polarity genes (SPG), which are also expressed in segmentally reiterated domains early in development that are aligned in defined anteroposterior positions within each segment [55, 68–70]. To our knowledge, a similar expression pattern of these three NK genes has not been reported from any arthropod species studied thus far, except for an early *Lbx* expression in *D. melanogaster* that does show an SPG-like pattern in both ectoderm and mesoderm [71]. Interestingly, *NK1*, *Lbx* and *Msx* are also expressed in an SPG-like pattern in the annelid *Platynereis dumerilii*, which shares with *E. rowelli* the formation of somites during its larval development [19]. Based on the distinct complementary expression of these genes early in development of *P. dumerilii*, the authors [19] have concluded that the role of the NK genes in anterior–posterior segment patterning might be conserved in protostomes. If so, one would expect these genes to be expressed in a similar anterior–posterior alignment in onychophorans.

However, besides *NK1*, *Lbx* and *Msx* at least two other NK cluster genes, namely *NK4* and *Tlx*, are expressed in an SPG-like pattern in *P. dumerilii* (Fig. 14B). Furthermore, despite the SPG-like expression patterns of *NK1*, *Lbx* and *Msx*, the relative positions and dimensions of individual domains within each somite clearly differ between *E. rowelli* and *P. dumerilii*. For example, while *NK1* and *Lbx* are expressed in the anterior and posterior halves of each somite in *E. rowelli*, respectively, both genes are co-expressed in the anterior portion of each somite in *P. dumerilii* (Fig. 14A, B). Likewise, *Msx* is expressed in the posterior two-thirds of each somite in *E. rowelli*, while it occupies the anterior portion of each somite in *P. dumerilii*. Most importantly, the expression of *Lbx* and *Msx* is restricted to the mesoderm in *E. rowelli*, whereas these genes show additional ectodermal domains in *P. dumerilii* (Fig. 14A, B). These major positional discrepancies suggest considerable differences in segment patterning mechanisms in these two taxa.

As another line of evidence, Saudemont et al. [19] state that the expression of *Lbx* is co-localized with the expression of *wingless* in *P. dumerilii* in a domain anterior to *engrailed*, which is similar to the situation in *D. melanogaster*, where *Lbx* expression is co-localized with and dependent on *wingless* expression. Based on these similarities, they consider it as unlikely that the complementary expression of *Lbx*, *wingless* and *engrailed* might have been recruited independently in annelids and arthropods [19]. In contrast to this, *Lbx* is not expressed in the ectoderm of *E. rowelli* at any developmental stage and, thus, is not co-localized with the exclusively ectodermal *wingless* expression [55]. Instead, the *Lbx* domain might partially overlap with the mesodermal *engrailed* domain in onychophorans, which is devoid of *Lbx* expression in *D. melanogaster* and *P. dumerilii*. Consequently, one would have to assume that this pattern has been modified to a large extent in onychophorans, while it was retained in annelids and arthropods. However, the argumentation of Saudemont et al. [19] is based on the expression of only a single NK gene in two distantly related species. Further data from other protostome taxa, including other annelids [72, 73] and arthropods [74–78], so far have not revealed any evidence for an involvement of NK genes in segment formation, which would indicate several independent losses of this pattern in many protostome taxa.

In summary, if the NK genes were involved in the anterior–posterior regionalization of segments in the protostome ancestor, as proposed by Saudemont et al. [19], one would expect a similar set of NK genes to be expressed in defined anteroposterior positions in most protostome taxa, similar to what has been described from the segment polarity genes *engrailed*, *hedgehog*, *wingless* and *cubitus interruptus* [55, 57, 68]. However, the considerable differences and the lack of specific similarities in the expression patterns observed in *P. dumerilii* and *E. rowelli*, as well as the absence of similar patterns in almost all protostomes studied thus far rather indicate that *NK1*, *Msx* and *Lbx* might have been recruited independently to fulfill similar functions in the regionalization of segments in annelids and onychophorans. On the other hand, even though these genes show a segmentally reiterated pattern in both species, which resembles an SPG-like expression, so far there is no convincing evidence for their potential role in the anterior–posterior regionalization of segments.

### Conserved expression patterns of NK cluster genes in the mesoderm of *E. rowelli*

One of the most intriguing features of the NK cluster genes is their seemingly conserved expression in mesodermal derivatives across bilaterians, including various somatic muscles [6, 7, 18]. For example, *NK1*, *Msx* and *Lbx* are expressed in non-overlapping patterns in different sets of longitudinal and parapodial muscles in the annelid *P. dumerilii*, suggesting that their transcripts might provide identity information for the differentiation of these muscles [19]. In the fruit fly *D. melanogaster*, *NK1*, *Msx* and the two *Lbx* orthologs (*lbl* and *lbe*) are mainly expressed in subsets of muscle founder cells, including the developing dorsal body wall muscles, lateral and segmental muscles [18, 37, 71, 77, 79]. In vertebrate embryos, including the mouse *Mus musculus* and the chicken *Gallus gallus*, the *Msx* and *Lbx* orthologs are expressed in specific sets of muscle precursors that will give rise to the limb musculature [38–41, 80–82].

Similarly, our data revealed that *NK1*, *NK3*, *NK4*, *NK5*, *Lbx* and *Tlx* are expressed in distinct, mesodermal domains in the developing limbs in embryos of *E. rowelli*. *NK1*, *NK4* and *Lbx* show a similar pattern in each developing leg, which follows an anterior-to-posterior progression in development. Similar *Tlx* domains appear in all developing limbs, although they differ in size and shape in the developing jaws and slime papillae, which are modified cephalic appendages and show a derived muscle arrangement ([56, 83]; see also Fig. 9A–C in Ref. [84]).

These findings indicate that the mesodermal domains of *NK1*, *NK4*, *Lbx* and *Tlx* might correspond in position to the individual developing limb muscles, and that these genes might provide positional identity information for the limb muscles in onychophorans. However, since development of the somatic musculature of onychophorans has not been studied and since the exact number and arrangement of individual leg muscles has not yet been clarified [85–90], the relation of these expression patterns to the development of specific sets of muscles in the onychophoran limbs remains unclear. Nevertheless, our results indicate that the involvement of NK genes in the development and differentiation of somatic musculature might be conserved in Bilateria [19], or at least Nephrozoa, since comparative NK gene expression data are missing from Xenacoelomorpha, the sister group of Nephrozoa [16].

In contrast to the mostly similar mesodermal expression patterns of *NK1*, *NK4*, *Lbx* and *Tlx*, the posterior domains of *NK3* and *NK5* are elongated in the fourth and fifth developing legs with respect to their corresponding domains in the remaining limbs. Compared to the jaws and slime papillae, however, these limbs do not show a derived muscle arrangement that would explain these differences [56]. Interestingly, these patterns resemble the expression of *odd*-*skipped*, *pox*-*neuro* and *pax3/7*, which also show enlarged domains in the fourth and fifth legs [11, 68]. This pattern might correspond to the anlagen of specific types of nephridia along the onychophoran body: The small *NK3* domains in the first three leg-bearing segments might be localized in the tiny nephridial anlagen of these segments, while the elongated *NK3* and *NK5* domains in the fourth and fifth leg-baring segments might be associated with the large, specialized “labyrinth organs” developing in these segments ([91–93]; see also Fig. 10A in [94]). This striking pattern indicates that these genes might be involved in the development of nephridia and their derivatives in Onychophora. To our knowledge, a comparable role of NK genes in nephridiogenesis has only been reported from the annelid *P. dumerilii* thus far [19].

**Fig. 15:**
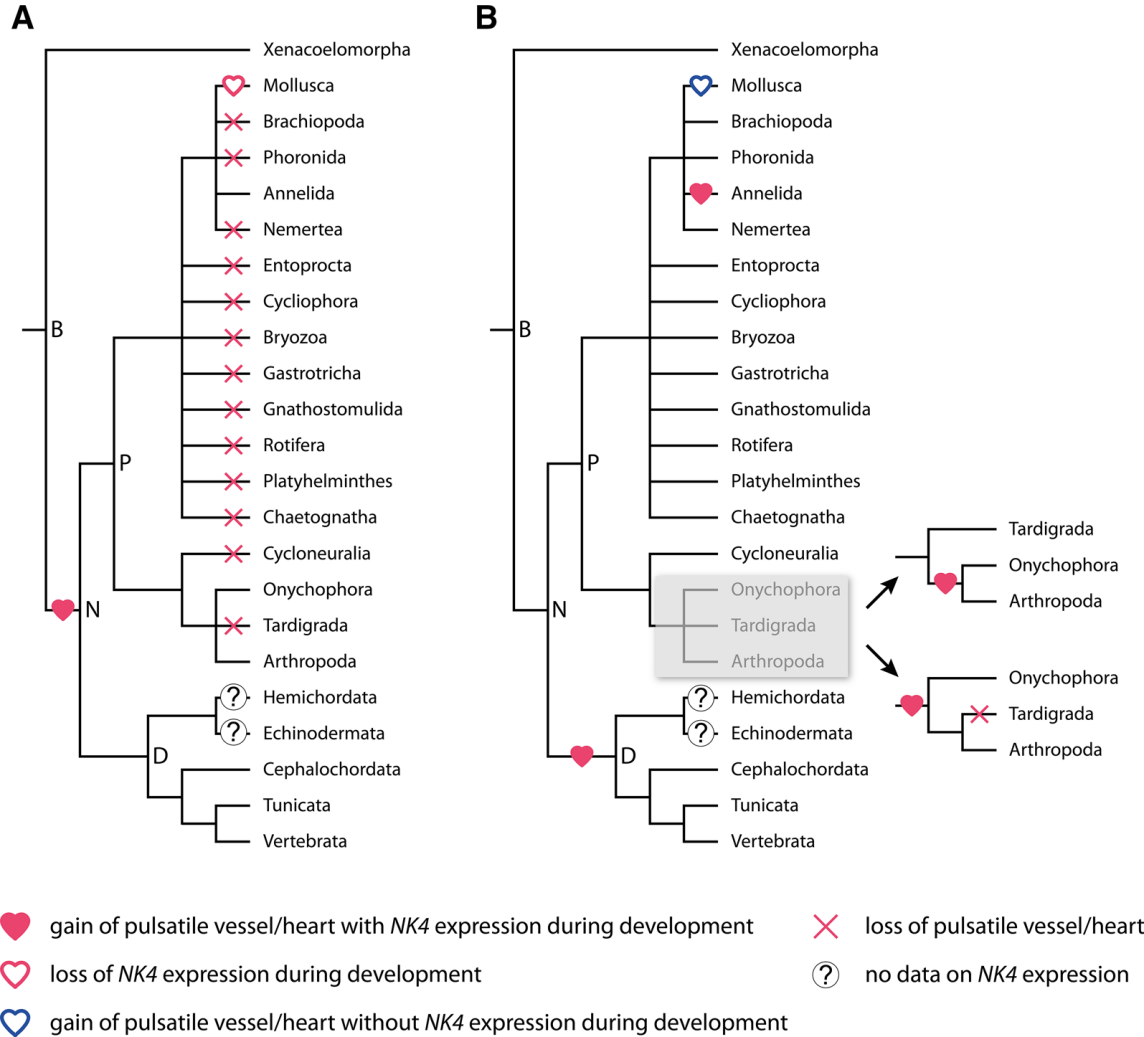
Alternative scenarios on the evolution of pulsatile organs in nephrozoans. Phylogeny modified from Ref. [16]. **A** Single origin of the heart and expression of *NK4* in cardiac tissue in the last common ancestor of Nephrozoa. According to this scenario, multiple independent losses of hearts occurred in numerous spiralian and ecdysozoan taxa. Additionally, expression of *NK4* in cardiac tissue was lost in mollusks. **B** Convergent evolution of hearts in deuterostomes, panarthropods, annelids and mollusks with either a gain of heart in the onychophoran/arthropod lineage or a secondary loss of the heart in tardigrades. According to this scenario, *NK4* might have been recruited independently to fulfill regulatory functions during heart development in vertebrates, panarthropods and annelids, but not in mollusks. Abbreviations: B, Bilateria; D, Deuterostomia; N, Nephrozoa; P, Protostomia

Apart from the expression of NK genes in the developing limb muscles, we observed peculiar mesodermal stripes of *NK4* expression along the dorsal rim of the lateral blastoderm bands. The position of this expression might correspond to mesenchymal cells of the dorsal coelomic linings that move into the space above the midgut to form the future heart [56, 95–97]. This pattern strongly resembles the previously described expression of the T-box gene *H15* in the dorsal tube of the onychophoran *Euperipatoides kanangrensis* [98]. This, in turn, is reminiscent of the overlapping expression of *NK4* and *H15* in the beetle *Tribolium castaneum*, the fly *D. melanogaster* and the spider *Cupiennius salei* along the dorsal rim of the lateral germ bands in cells that have been identified as heart precursors in these animals, suggesting that this pattern might be conserved in onychophorans and arthropods [18, 28–30, 35]. These similarities support the hypothesis that the panarthropod hearts are homologous. In contrast to onychophorans and arthropods, however, a vascular system with a pulsatile organ is absent from the third major panarthropod taxon, the tardigrades. This absence might be a secondary loss due to the miniaturized body of tardigrades [99]. Depending on the phylogenetic position of tardigrades, our data suggest that the last common ancestor of either panarthropods or onychophorans plus arthropods possessed a pulsatile dorsal vessel that expressed *NK4* during development (Fig. 15A, B).

Interestingly, studies of *NK4* expression in other bilaterians, including annelids and chordates, revealed similar patterns in the pulsatile dorsal vessel or heart, which has led to the hypothesis that a role in the formation of the pulsatile dorsal vessel or heart might be conserved among bilaterians, or at least nephrozoans, since xenacoelomorphs do not possess a circulatory system [19, 100–102]. Thus, the “urnephrozoan” might have already possessed a pulsatile dorsal vessel. A possible conservation of the *NK4* expression in heart precursor cells and a common origin of the heart in panarthropods or onychophorans plus arthropods might support this hypothesis. However, many protostome taxa, including cycloneuralians and numerous spiralian/lophotrochozoan taxa, do not possess heart-like organs or pulsatile vessels. If a heart-like structure indeed evolved only once in the nephrozoan ancestor, one would have to assume multiple independent losses of pulsatile organs in these taxa. Like tardigrades, many of these taxa show extensive miniaturization [103]. Since it has been shown that miniaturization often results in the reduction or complete loss of organs or entire organ systems, multiple independent losses of the vascular system including heart-like structures seem possible [103]. In contrast to this, *NK4* expression data from the cephalopod mollusk *Sepia officinalis* revealed that *NK4* is not involved in the formation of the heart but rather in somatic muscle development in this species [104]. Consequently, if one assumes a single origin of the heart in the nephrozoan ancestor, mollusks would have lost *NK4* expression in cardiac tissue but retained the heart itself (Fig. 15A).

Interestingly, the example of *S. officinalis* shows that *NK4* is not essential for proper heart development and that the formation of pulsatile tissue can be mediated by other molecular mechanisms [104]. The expression of *NK4* in the somatic musculature in *S. officinalis* rather indicates an ancestral role of this gene in somatic muscle development. Thus, an alternative scenario of heart evolution in nephrozoans is conceivable (Fig. 15B). According to this scenario, a heart was absent in the last common ancestor of nephrozoans and pulsatile organs evolved four times independently in deuterostomes, panarthropods (or onychophorans plus arthropods), annelids, and mollusks. According to this scenario, *NK4* might have been involved in somatic muscle development in the nephrozoan ancestor and was recruited independently to fulfill major regulatory functions during heart development in annelids, panarthropods and deuterostomes but not in mollusks.

### Non-regionalized neural expression of NK cluster genes and the NKL gene *NK2.2* in *E. rowelli* supports convergent evolution of bilaterian nerve cords

In addition to mesodermal domains, we observed an expression of *NK3*, *NK5*, *NK6.1*, *NK6.2*, *Lbx*, *Msx* and *NK2.2* in the developing nervous system of the onychophoran embryo. While *NK3*, *NK5*, *Msx* and *NK2.2* are expressed in the anlagen of both the brain and the ventral nerve cords, the expression of the two *NK6* copies is restricted to the ventral nervous system, and *Lbx* is confined to the developing brain. Interestingly, an involvement of *NK5*, *NK6*, *Msx*, *Lbx*, *Tlx* and *NK2.2* in neural development has also been reported from other bilaterians, including arthropods, annelids and vertebrates [18, 19, 44, 105]. This supports the assumption that these genes were involved in neural development in the last common bilaterian ancestor [18, 19, 44].

**Fig. 16:**
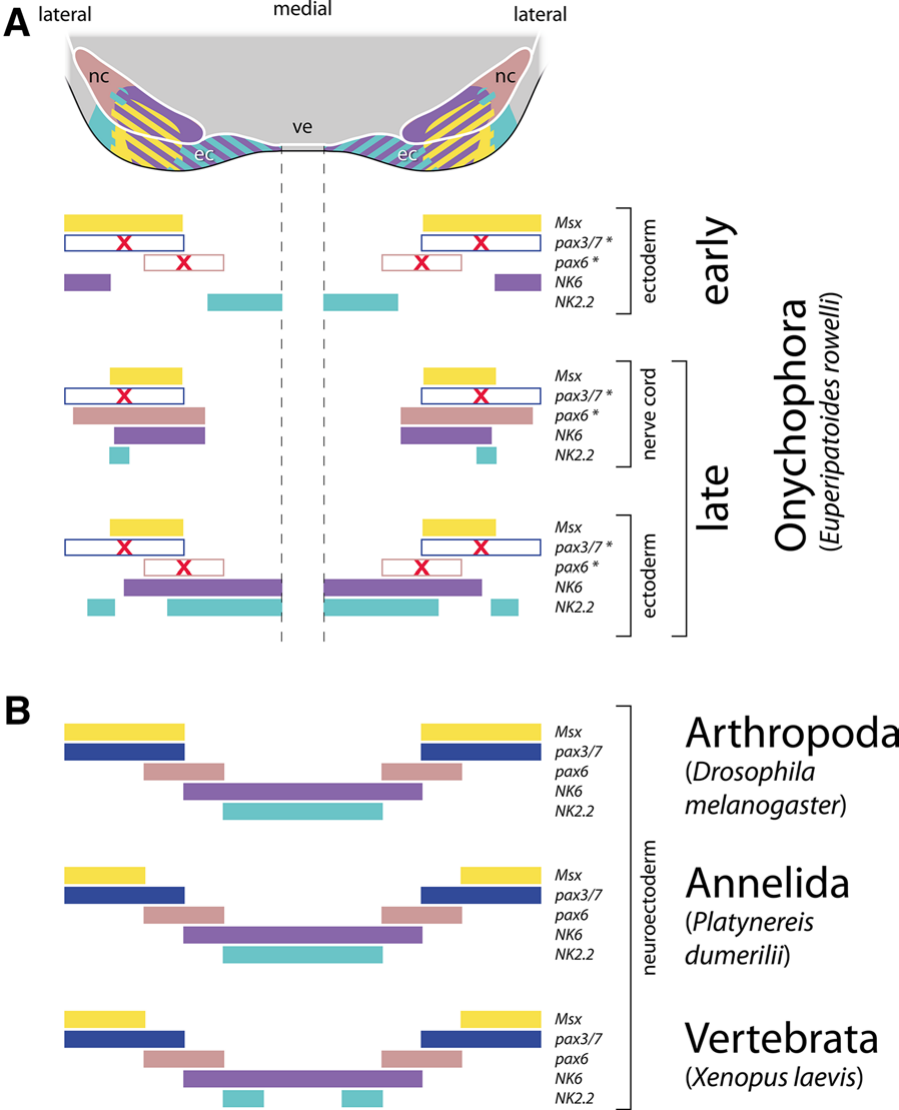
Comparison of the expression of mediolateral patterning genes in onychophorans, arthropods, annelids and vertebrates. **A** Schematic drawing of the ventral ectoderm and nerve cords of a late stage embryo of *E. rowelli* showing expression of *Msx*, *pax3/7*, *pax6*, *NK6* and *NK2.2*. Bars below show the mediolateral extent of each domain in the ventral ectoderm and nerve cords in early and late developmental stages. Note that the ectodermal expression is interrupted by the ventral extra-embryonic tissue. Empty bars with red crosses indicate the presumed absence of expression in the nerve cord and ventral ectoderm. Asterisks indicate expression data described in Refs [11, 68, 107]. **B** Expression of *Msx*, *pax3/7*, *pax6*, *NK6* and *NK2.2* in the neuroectoderm of the fruit fly *Drosophila melanogaster*, the annelid *Platynereis dumerilii* and the vertebrate *Xenopus laevis*. Abbreviations: ec, ventral ectoderm; nc, nerve cord; ve, ventral extra-embryonic tissue. Diagrams in B modified from Ref. [106]. See also Additional file 4 for a comparison of *Msx*, *pax6*, *NK6* and *NK2.2* domains in *E. rowelli*

In the trunk of *E. rowelli*, *NK5*, *NK6.1*, *NK6.2*, *Msx*, *Lbx*, *Tlx* and *NK2.2* are expressed in uniform domains along the body. However, *NK2.2*, *Msx* and both copies of *NK6* show peculiar mediolaterally regionalized patterns (Fig. 16A). While *NK2.2* is expressed in parallel, continuous, medial and lateral stripes along the body, *Msx* and both copies of *NK6* show both, continuous bands of expression within each nerve cord as well as segmentally reiterated domains in the medial ectoderm (Fig. 16A). Interestingly, *Msx*, *NK6* and *NK2.2* have been reported to be involved in the mediolateral regionalization of the nervous system in vertebrates, the fly *D. melanogaster* and the annelid *P. dumerilii* [19, 44, 46, 105]. This regionalization is mediated by the staggered expression of the transcription factors NK6, Msx, NK2.2, Pax6 and Pax3/7 in the neuroectoderm of these animals (Fig. 16B), thus defining a specific mediolateral arrangement of neurogenic domains and neuron types [19, 44, 105]. While the medial *NK2.2*^+^/*NK6*^+^ domain gives rise to a medial column of serotonergic neurons, the adjacent *NK6*^+^/*pax6*^+^ area forms an intermediate column of cholinergic motor neurons and the lateral *pax6*^+^/*pax3/7*^+^ and *pax3/7*^+^/*msx*^+^ domains establish a lateral column of interneurons and lateral sensory trunk neurons [17, 44]. This seemingly conserved mediolateral patterning has been used as a major argument for proposing an ancestral condensed, mediolaterally patterned ventral nerve cord in the “urbilaterian” [17, 44, 105]. The absence of a mediolateral patterning of these genes in hemichordates, nematodes and planarians has been interpreted as independent losses in these lineages [44].

However, recent studies of representatives of Xenacoelomorpha revealed that *NK6*, *Msx*, *NK2.2*, *pax6* and *pax3/7* expression is unrelated to the trunk neuroanatomy in these taxa, suggesting that the mediolateral patterning evolved after the Xenacoelomorpha–Nephrozoa split [17]. Interestingly, investigation of these genes in additional nephrozoan taxa, including Rotifera, Nemertea, Brachiopoda and Enteropneusta, revealed that a mediolateral regionalization is also absent or only partially present in these taxa [17, 42]. Moreover, despite the presence of mediolateral regionalization in the annelid *P. dumerilii* (Errantia, Phyllodocida), there is no evidence for such a pattern in the annelid *Owenia fusiformis* (Sedentaria, Sabellida), although its trunk neuroanatomy largely resembles that of *P. dumerilii*, *D. melanogaster* and vertebrates [17]. Thus, two possible scenarios on the evolution of condensed medial nerve cords among bilaterians have been proposed ([17] but see an opposing view in Ref. [106]). According to the first scenario, the mediolateral patterning of the central nervous system in vertebrates, *D. melanogaster* and *P. dumerilii* is homologous, thus reflecting the ancestral bilaterian or nephrozoan state, which would imply multiple independent losses or modifications. Alternatively, the absence of mediolateral regionalization of the central nervous system in xenacoelomorphs, many spiralians and some annelids might indicate a convergent evolution of this patterning system in vertebrates, arthropods and some annelids.

Our results show that *NK2.2*, *NK6.1*, *NK6.2* and *Msx* are expressed in dynamic patterns early and late in development of *E. rowelli*. At the onset of neurogenesis, when neural precursors segregate from the ventral ectoderm [56], expression of these genes is mainly restricted to the ventral ectoderm (Fig. 16A). *NK2.2* and *Msx* are expressed in non-overlapping medial and lateral domains, respectively, that resemble the *NK2.2* and *Msx* domains of other bilaterians. In contrast to this, *NK6* is expressed in a lateral domain, overlapping with *Msx*, but not with *NK2.2* (Fig. 16A). This is different from what has been reported from arthropods, annelids and vertebrates, where *NK6* is expressed in a medial domain, overlapping with *NK2.2* but not with *Msx* (Fig. 16B).

Later in development, after most neural precursors have been segregated and the developing nerve cords have delaminated from the ventral ectoderm, these genes are expressed in both the ventral ectoderm and the nerve cords where they show different patterns (Fig. 16A). In the ventral ectoderm, *NK2.2* and *NK6* are expressed in medially restricted, largely overlapping domains, while *Msx* expression is confined to the central region, partially overlapping with the medial *NK2.2* and *NK6* domains. *NK2.2* shows an additional domain in the lateral ectoderm, which partially overlaps with the *Msx* domain, but not with the medial *NK6* domain. In the nerve cords, *NK6* and *Msx* are expressed in broad, largely overlapping domains that are confined to the medial part, while *NK2.2* is restricted to a spot-like expression in the lateral nerve cord, overlapping with the *NK6* and *Msx* domains (Fig. 16A).

Similarly, previous gene expression data on *pax6* and *pax3/7* did not provide any evidence for regionalized mediolateral patterning in Onychophora [11, 107]. Instead, *pax6* is expressed in a broad domain, which covers the entire width of the nerve cord in late developmental stages (see Fig. 9D in [11]), thus overlapping with the *NK2.2*, *NK6* and *Msx* domains and extending further laterally (Fig. 16A). In contrast, *pax3/7* is not expressed in the developing nerve cords at any developmental stage (see Fig. 8A–D in [11] and Fig. 5A–C in [68]). Thus, irrespective of the developmental stage studied, neither our results nor previously published data on *pax6* and *pax3/7* expression provide any evidence for the presence of adjacent *NK2.2*^+^/*NK6*^+^, *NK6*^+^/*pax6*^+^, *pax6*^+^/*pax3/7*^+^ and *pax3/7*^+^/*msx*^+^ columns in the onychophoran nerve cords.

These results are in line with selective stainings of specific neurons, including retrograde fills of the leg nerves, and immunolabeling of various neurotransmitters and neuromodulators, which did not provide any evidence for adjacent columns of medial serotonergic neurons, cholinergic motor neurons, lateral interneurons or sensory neurons in the onychophoran nerve cords [108–110]. Instead, most of the neuron types are located in largely overlapping areas [108]. For example, the somata of serotonergic neurons are not restricted to a medial area but are rather distributed in a random fashion in the ventromedial and ventral perikaryal layers [108, 109].

While the expression of *NK2.2*, *NK6* and *Msx* in the ventral ectoderm of early developmental stages as well as in the nerve cords of late developmental stages might be correlated to the specification of neurons, their corresponding patterns in the ventral ectoderm of late developmental stages are not necessarily correlated with any neural structures. Interestingly, the double-paired patterns of *NK6* and *Msx* in the ventromedial ectoderm correspond to the emergence of paired ectodermal thickenings which are the anlagen of the ventral and preventral organs [84, 94, 111]. Although these structures arise from the ventral ectoderm, they do not seem to contribute any cells to the central nervous system but rather persist as attachment sites for segmental limb muscles in adult onychophorans ([56, 84, 112, 113] but see ref [114] for an opposing view on a putative function of ventral organs in neurogenesis). Interestingly, similar patterns have been reported from *Delta* and *Notch* transcripts that are expressed in two pairs of bilaterally symmetric domains on the ventrum of each segment in embryos of *Euperipatoides kanangrensis* and *E. rowelli* [84, 114, 115]. This characteristic, double-paired pattern appears only after most neurons have been segregated from the neuroectoderm [84, 109, 116]. Thus, it has been concluded, that these *Delta* and *Notch* domains specify regions of the ectoderm that give rise to the ventral and preventral organs rather than neurogenic tissue in the onychophoran embryo [56, 84]. Likewise, our results indicate that the double-paired segmental *Msx* and *NK6* domains in the ventral ectoderm might provide identity information for specific regions of the ectoderm that give rise to the ventral and preventral organs rather than neurogenic tissue because the nerve cords have already been segregated from the ectoderm at this developmental stage (cf. Ref. [56]).

**Fig. 17:**
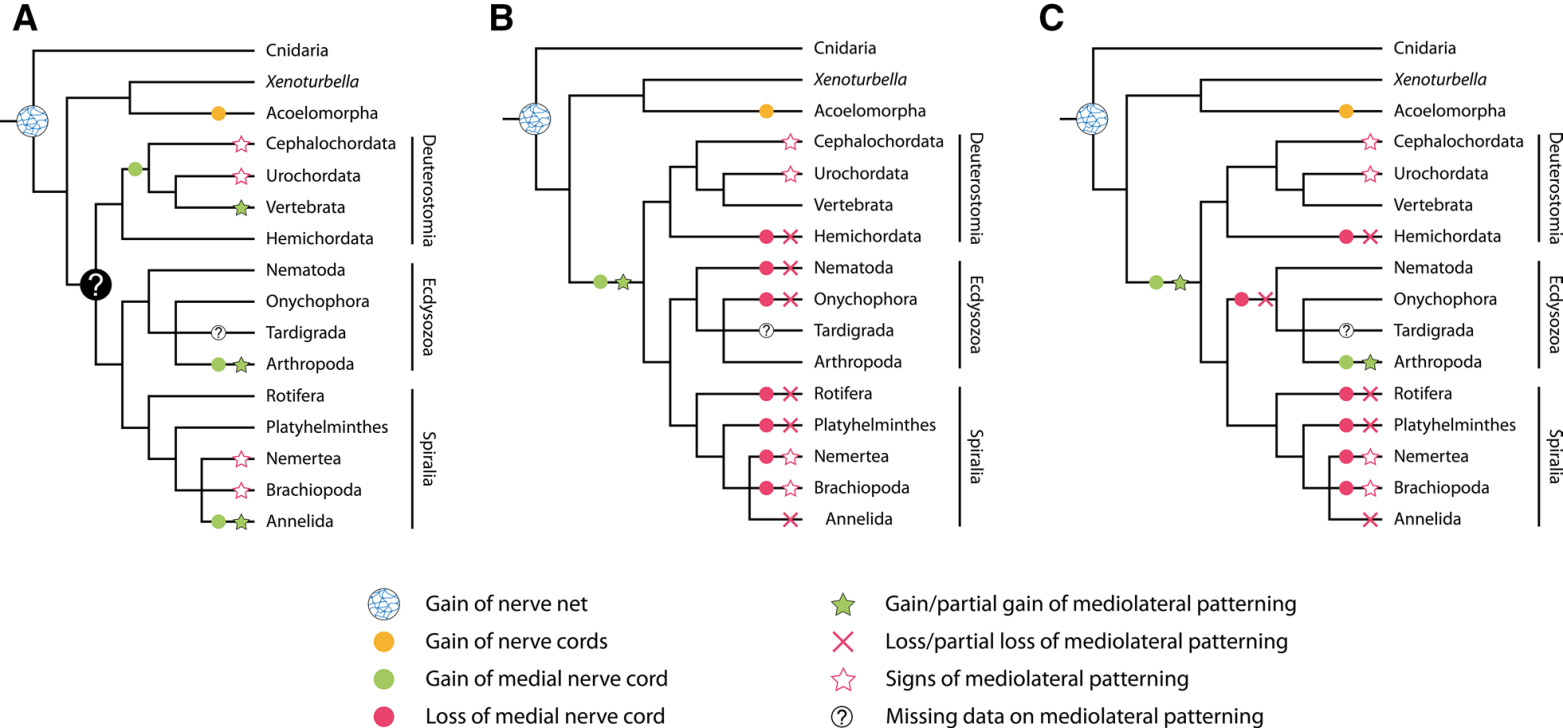
Alternative scenarios on the evolution of mediolateral patterning of nerve cords among bilaterians. Scenarios A and B are modified from Ref. [17]. **A** Convergent evolution of similarities of the mediolateral patterning in vertebrates, arthropods and some annelids. The reconstruction of the ancestral morphology of the nervous system in the “urnephrozoan” is still unresolved due to the diversity of nerve cord arrangements in the nephrozoan lineages (white question mark). **B** The “urnephrozoan” already possessed a medially condensed nerve cord and mediolateral patterning. Thus, the medially condensed nerve cords of vertebrates, arthropods and annelids are homologous, implying multiple losses of mediolateral patterning. **C** The “urnephrozoan” already possessed a medially condensed nerve cord and mediolateral patterning. The medially condensed nerve cords of vertebrates and annelids are homologous. As a result, multiple independent losses of mediolateral patterning would have occurred, including the ecdysozoan ancestor, which would imply an independent evolution of medially condensed nerve cords in arthropods

Thus, *NK6*, *Msx*, *NK2.2*, *pax6* and *pax3/7* expression seems to be unrelated to the trunk neuroanatomy in onychophorans, which is similar to what has been reported from Xenacoelomorpha, Rotifera, Nemertea, Brachiopoda and Enteropneusta, and some Annelida [17]. Consequently, if a complex medial nerve cord with a distinct regionalization of specific neuron types was present in the nephrozoan ancestor, one would have to assume either multiple losses of the mediolateral pattern in several lineages (including nematodes and onychophorans; Fig. 17B), or a single loss in the ecdysozoan ancestor followed by a secondary gain of this pattern in the arthropod lineage (Fig. 17C). The latter scenario would further imply a homology of the medially condensed nerve cords in vertebrates and annelids, but also an independent evolution of medially condensed nerve cords in arthropods (Fig. 17C). Alternatively, a mediolateral regionalization of the nerve cords might have never existed in the panarthropod ancestor, which would support the hypothesis [17] of convergent evolution of a condensed, mediolaterally patterned nerve cord in vertebrates, arthropods and some annelids (Fig. 17A).

## Conclusion

Our analysis of the NK cluster genes in the onychophoran *E. rowelli* revealed that these genes are involved in various developmental processes. Our major findings can be summarized as follows:

- *NK1*, *Msx* and *Lbx* are expressed in a segment polarity-like pattern early in development of *E. rowelli*—a pattern that otherwise has only been reported from the annelid *P. dumerilii*. However, major differences in timing, position and extent of these patterns suggest considerable differences in segment patterning mechanisms between the two animal groups, which argue against a common origin of the segment polarity patterns of NK genes in onychophorans and annelids.
- *NK1*, *NK3*, *NK4*, *NK5*, *Lbx* and *Tlx* are expressed in distinct domains in the developing limb musculature of *E. rowelli*, which corresponds to the involvement of these genes in somatic muscle development in other bilaterian taxa, suggesting that this role is conserved in Nephrozoa.
- Our results on *NK4* expression further provide evidence for a common origin of the dorsal heart in onychophorans and arthropods.
- *NK3*, *NK5*, *NK6*, *Lbx*, *Msx* and *NK2.2* are expressed in the developing nervous system of *E. rowelli*, which resembles the situation in other nephrozoans, indicating that these genes were involved in neural patterning in the last common ancestor of Nephrozoa. Furthermore, our results indicate that a staggered mediolateral expression of *NK6*, *Msx* and *NK2.2* was either lost or was never present in Onychophora.

## Methods

### Specimens and sample preparation

Specimens of *Euperipatoides rowelli* Reid, 1996 (Peripatopsidae) were collected from decaying logs in the Tallaganda State Forest (35°26′S, 149°33′E, 954 m, New South Wales, Australia) in October 2011, January 2013 and October 2016. Permissions for specimen collection were obtained from the National Parks & Wildlife Service New South Wales (permit numbers: SL100159 and SL101720). Permissions for the export of specimens were obtained from the Department of Sustainability, Environment, Water, Population and Communities (permit numbers: PWSP104061, PWSP208163, and PWS2016-AU-001023). The animals were maintained in the laboratory as described previously [118]. Females were anesthetized with chloroform vapor for 10–20 s and the reproductive tracts were dissected and transferred to a physiological saline [119]. Embryos were dissected from the uteri, and the two membranes surrounding each embryo were removed using fine forceps. The embryos were staged according to Walker MH and Tait NN [53] with modifications described previously [55, 120]. After staging, the embryos were fixed overnight in 4% paraformaldehyde in phosphate-buffered saline (PBS; 0.1 M, pH 7.4), dehydrated in a graded methanol series and stored in absolute methanol at − 20 °C.

### Identification of NK homologs

Library preparation and assembly of the embryonic transcriptomes from the onychophoran *E. rowelli* and the tardigrade *Hypsibius exemplaris* were performed as described previously [121, 122]. The latter species was commonly referred to as “*Hypsibius dujardini* (Doyère, 1840)” in the literature (e.g., [51, 67, 122–126]), but it was described recently [127] as *Hypsibius exemplaris* Gąsiorek et al., 2018. To identify the expressed homologs of NK homeobox genes in onychophorans and tardigrades, the transcriptome libraries of *E. rowelli* and *H. exemplaris* were screened by local tBLASTx searches [128]. For the gene *Tlx*, the previously published homolog *clawless* from *Euperipatoides kanangrensis* was used as a query [129]. For all other NK genes, previously published sequences from *Drosophila melanogaster* were retrieved from the online database HomeoDB (http://homeodb.zoo.ox.ac.uk/; accessed 25 Jan 2018) [130, 131] and used as queries. The retrieved putative NK gene sequences were then verified by reciprocal BLASTn and tBLASTx searches against the nr database of GenBank. Additional BLAST searches of the putative tardigrade NK homologs in two independently published genomes of *H. exemplaris* [see 50, 51] yielded nearly identical sequences. The sequences of identified NK homeobox genes from *E. rowelli* were made available in GenBank (Table 2).

**Table 2:** GenBank accession numbers of the identified NK homeobox genes from *E. rowelli*, and commonly used synonyms for each gene family

Gene name	Accession number	Synonyms
*NK1*	MH036363	*slouch (slo), S95, Nkx1, HSPX153, SAX, ceh*-*1*
*NK3*	MH036364	*bagpipe (bap), BAPX, nkx3*
*NK4*	MH036365	*tinman (tin), Nkx2.3, Nkx2*-*6, Nkx2.5, Csx, Nkx2.7, ceh*-*28*
*NK5*	MH036366	*Hmx, H6, Nkx5, SOHO*-*1, mls*-*2*
*NK6.1*	MH036367	*Nkx6, Hgtx, Nnk6, Gtx, cog*-*1*
*NK6.2*	MH036368	
*Msx*	MH036369	*Drop (Dr), msh, vab*-*15*
*Lbx*	MH036370	*ladybird early (lbe), ladybird late (lbl), Hpx*-*6*
*Tlx*	MH036371	*clawless (cll), C15, 93Bal, Ect5, HOX11, Tcl3, Ncx, Enx, Rnx*
*NK2.2*	MH170042	*ventral nervous system defective (vnd), Nkx2*-*2/2*-*8/2*-*9, ceh*-*22*

### Sequence alignment, phylogenetic analysis and short read mapping

The amino acid sequences of the homeodomain of NK genes of various model organisms were retrieved from the online database HomeoDB (http://homeodb.zoo.ox.ac.uk/; accessed 25 Jan 2018) [130, 131]. Furthermore, publicly available resources and databases (nr, TSA and EST databases of GenBank) as well as the genomes of the centipede *Strigamia maritima* [58], the common house spider *Parasteatoda tepidariorum* [132], the tardigrade *Ramazzottius varieornatus* [52] and the crustacean *Daphnia pulex* [133] were screened for putative NK homologs. The previously published NK sequences of the polychaete *Platynereis dumerilii* were retrieved from GenBank [19]. For the phylogenetic analysis, we generated a dataset with a total of 382 sequences containing the 60 amino acid positions of the homeobox of various metazoans using the online tool MAFFT version 7 [http://mafft.cbrc.jp/alignment/server/; 134] with the L-INS-I alignment strategy (Additional file 5). Afterward, a maximum likelihood analysis was performed using the Pthreads-SSE3 version of RAxML v8.2.10 [135] under a dataset-specific GTR substitution model. The best tree was obtained from 100 independent inferences and GAMMA correction of the final tree. Bootstrap values were calculated from 1000 pseudoreplicates using the rapid bootstrapping algorithm implemented in RAxML (Fig. 1 and Additional file 6). The phylogeny was visualized with iTol and edited with Adobe (San Jose, CA, USA) Illustrator CS 5.1.

The abundance of the obtained NK homologs was estimated using segemehl v0.1.7 [136] by mapping the raw sequence reads of various embryonic transcriptome libraries of *E. rowelli* [see 121] back to the NK sequences of *E. rowelli* allowing for a maximum of 5% mismatching nucleotides per read. Afterward, the relative abundance [measured in matched nucleotides per position and per giga base pair (Gb)] of the NK genes were normalized using the relative abundance of the ribosomal protein RPL31 as a reference (= 100%) and visualized as a bar graph (Fig. 3; Additional file 2). We have chosen *RPL31* as reference since this gene shows the lowest variation [relative standard deviation (RSD) = 7.69%] among the studied transcriptomes as well as the tested putative reference genes (*GAPDH* and 44 ribosomal protein genes; Additional file 2). Furthermore, we analyzed the localization and orientation of NK genes in the publicly available genomes of *Hypsibius exemplaris* [see 50] and *Ramazzottius varieornatus* [see 52]. We checked the scaffold location, direction, distance as well as the number of other genes between each NK gene.

### Nomenclature

The nomenclature of NK genes is confusing since a large variety of synonyms has been established for each NK gene in different animal groups. Thus, we decided to use the general gene family names to avoid further confusion (see table 1 in Ref. [7], table 2 in Ref. [21], and table 1 in Ref. [137] for summaries of NK family genes and their synonyms). The abbreviation “*cll*” was used previously [129] for the *Tlx* ortholog in the onychophoran *Euperipatoides kanangrensis*; we instead use the more general name/abbreviation “*Tlx*” for the corresponding ortholog in the closely related species *E. rowelli*.

### RNA isolation, amplification of gene fragments, molecular cloning, probe preparation and whole-mount in situ hybridization

RNA isolation and cDNA synthesis were performed as described previously [11, 55, 138]. Fragments of *NK1*, *NK3*, *NK4*, *NK5*, *NK6*, *Msx*, *Lbx* and *Tlx* were amplified from the cDNA using specific primers (Table 3) and Phusion Green High-Fidelity DNA Polymerase (2 U/µl; Thermo Scientific, Waltham, MA, USA) to obtain PCR fragments with blunt ends. The gene fragments that were amplified from the cDNA (Table 3) were cloned into the pJet1.2/blunt cloning vector (Thermo Scientific). Digoxigenin-labeled RNA probes were prepared using the DIG RNA Labeling Kit SP6/T7 (Roche, Mannheim, Germany). In situ hybridization on whole embryos was performed as described previously [120] with the following modifications: (1) Stage V and VI embryos were digested in 25 µl of a chitinase/chymotrypsin solution (50 mg/ml chitinase and 15 mg/ml chymotrypsin in potassium phosphate buffer; Sigma-Aldrich, St. Louis, MO, USA) diluted in 1 ml PBST (PBS; 0.1 M; pH 7.4; with 0.1% Tween-20) prior to the pre-hybridization, followed by three 5 min washes in PBST; (2) after the staining reaction in NBT/BCIP solution and the postfixation with 4% PFA, the embryos were dehydrated in an increasing ethanol series (25%, 50%, 75%, 2 × 100% in PBST) and left in ethanol overnight; after rehydration in a decreasing ethanol series, the embryos were counterstained with the DNA-selective fluorescent dye 4′,6-diamidin-2-phenylindol (DAPI; Thermo Scientific, formerly Invitrogen; diluted 1:1000 in PBS) for 1 h and stored in 70% glycerol. Cross and sagittal sections were prepared manually using a razor blade.

**Table 3:** Specific primers used to amplify the transcripts of NK homeobox genes from cDNA of the onychophoran *E. rowelli*

Gene	Fragment length (in bases)	Direction	Primer sequence	Annealing temperature (°C)
*NK1*	730	Forward	ATGTTGGTGTATTGCCGGAGG	62
		Reverse	GTAGGCAAACTTGTGACTGGG	
*NK3*	649	Forward	TCTAAGCGACACTCTCACAGC	62
		Reverse	AGGAAGGATACATGACAGGCC	
*NK4*	900	Forward	TAAAGATATTTTAAATCTTAGCGAACAAG	58
		Reverse	GCCACATATTATTTACACAGTGACATC	
*NK5*	611	Forward	CCATCGACTCAAGTTCCAACG	62
		Reverse	ATGTTTGCTGCCTCCATCTCG	
*NK6*	656	Forward	GACTACACCAACCCAAACACG	62
		Reverse	TGGAACCATACCTTGACCTGG	
*Msx*	712	Forward	TTGCAGGCGAGTAGTAACTCG	62
		Reverse	ATGACAGTCCTAAAGCAGGCG	
*Lbx*	579	Forward	CGAAGAGCATTTGAAAGCGC	58
		Reverse	GTCACGTGTATTCGTGTAGC	
Tlx*	703	Forward	GCTAGAGACAATTCTCC	43
		Reverse	GAACTGTCCTCAGCCC	
*NK2.2*	486	Forward	ATGGCTCTAAATGGGTCAAAAAG	62
		Reverse	TGGTGGATCTCCTGCATTTG	

*Primer sequences obtained from Ref. [129]

Controls were performed using the sense probes of each gene and the same protocol as described above. Early embryonic stages (0 to IV) did not show any labeling. Staining artifacts that have been reported to appear in cuticular structures in late developmental stages [139, 140] are absent in the sense probes treated with chitinase/chymotrypsin prior to hybridization. The only remaining unspecific labeling appears in the sclerotized jaws and claws of stage VII embryos. Thus, we conclude that the staining obtained with the antisense probes is specific for all genes.

### Microscopy and image processing

The embryos were analyzed with the stereomicroscope Axio Zoom V16 (Carl Zeiss MicroImaging GmbH, Jena, Germany) equipped with an Axiocam 503 color digital camera (Carl Zeiss MicroImaging GmbH). Micrographs were taken at different focal planes and merged to single projections using the ZEN 2012 blue edition software version 1.1.2.0 (Carl Zeiss MicroImaging GmbH) or the Auto-Blend Layers function in Adobe Photoshop version CS 5.1. The micrographs were adjusted for brightness and contrast using Adobe Photoshop CS 5.1. Final panels and diagrams were designed using Adobe Illustrator CS 5.1.

## Additional files

**Additional file 1.** NK (blue) and NKL (orange) gene complements of different bilaterian species. Numbers indicate the number of genes, dashes indicate the absence thereof, numbers in brackets indicate the number of pseudogenes, question marks indicate missing data. The gene complements were retrieved from publicly available data as well as the sources specified below the table.

**Additional file 2.** Comparative transcriptomic analysis of NK genes in the onychophoran *E. rowelli* showing the average number of matched nucleotides per position and GB (upper table) of all studied NK genes from different transcriptome libraries of partially pooled embryonic stages, and the relative expression level in comparison to the abundance of ribosomal protein *RPL31* transcripts (lower table) obtained from the short read mapping analysis.

**Additional file 3.*** NK6.2* expression at consecutive developmental stages in embryos of the onychophoran *E. rowelli*. Developing limbs are numbered. Anterior is left in all images. **A** Stage II embryo in ventrolateral view. **B** Proctodeum of a stage II embryo in ventral view. Arrowheads indicate the expression around the proctodeum. **C** Stage III embryo in lateral view. Arrow indicates the decreasing intensity of the signal. **D** Developing jaw, slime papilla and first leg of a stage III embryo. Signals in the ventral ectodermal thickenings and ventral nerve cords in are indicated with asterisks and arrowheads, respectively. **E** Stage V embryo in ventral view. A weak expression is visible in the ventral nerve cords (arrowheads). Abbreviations: at, developing antenna; cl, cephalic lobe; de, dorsal extra-embryonic tissue; jw, developing jaw; js, jaw segment; po, proctodeum; sp, developing slime papilla; ss, slime papilla segment; ve, ventral extra-embryonic tissue. Scale bars: A, C: 200 µm; B, D: 100 µm: E: 500 µm.

**Additional file 4.** Cross sections of stage IV embryos of the onychophoran *E. rowelli* showing the expression of *NK6.1* (A, A’, B, B’), *Msx* (C, C’) and *NK2.2* (D, D’) in the ventral ectoderm (empty arrowheads) and developing nerve cords (filled arrowheads). Developing nerve cord is indicated with dashed lines, developing neuropil is indicated with asterisks. Scale bars: A–C’: 50 µm; D, D’: 100 µm.

**Additional file 5.** Alignment of the 382 NK homeodomains of various metazoan taxa that was used for the maximum-likelihood analysis.

**Additional file 6.** Maximum-likelihood analysis of the NK cluster and NKL genes among metazoans. The ML phylogeny was computed using the Pthreads-SSE3 version of RAxML v8.2.10.
