# Supplementary material for: Expression of NK cluster genes in the onychophoran Euperipatoides rowelli: implications for the evolution of NK family genes in nephrozoans
Source: EvoDevo. 2018 Jul 18;9:17. doi: 10.1186/s13227-018-0105-2 (PMC6050708; doi:10.1186/s13227-018-0105-2)
Supplement: Supplementary file 1 — Additional file 1. NK (blue) and NKL (orange) gene complements of different bilaterian species. Numbers indicate the number of genes, dashes indicate the absence thereof, numbers in brackets indicate the number of pseudogenes, question marks indicate missing data. The gene complements were retrieved from publicly available data as well as the sources specified below the table. [file 13227_2018_105_MOESM1_ESM.docx]

|  | ***Amphimedon queenslandica*** | ***Nematostella vectensis*** | ***Lottia gigantea*** | ***Platynereis dumerillii* ^2^** | ***Caenorhabditis elegans* ^1^** | ***Ramazzottius varieornatus* ^4^** | ***Hypsibius exemplaris* ^5^** | ***Euperipatoides rowelli*** | ***Parasteatoda tepidariorum*** | ***Strigamia maritima* ^3^** | ***Daphnia pulex* ^6^** | ***Tribolium castaneum* ^1^** | ***Apis mellifera* ^1^** | ***Drosophila melanogaster* ^1^** | ***Branchiostoma floridae* ^1^** | ***Xenopus tropicalis* ^1^** | ***Gallus gallus* ^1^** | ***Danio rerio* ^1^** | ***Mus musculus* ^1^** | ***Homo sapiens* ^1^** |
| --- | --- | --- | --- | --- | --- | --- | --- | --- | --- | --- | --- | --- | --- | --- | --- | --- | --- | --- | --- | --- |
| ***NK1*** | — | 1 | 2 | 1 | 1 | 1 | 1 | 1 | 2 | 1 | 1 | 1 | 1 | 1 | 2 | 3 | 2 | 2 | 2 | 2 |
| ***NK3*** | — | 2 | — | 1 | — | 1 | 1 | 1 | — | 1 | — | 1 | 1 | 1 | 1 | 3 | — | 3 | 2 | 2 |
| ***NK4*** | 1 | 1 | 1 | 1 | 1 | 1 | 1 | 1 | 1 | 1 | — | 1 | 1 | 1 | 1 | 3 | 2 | 3 | 3 | 3 |
| ***NK5*** | — | 1 | 1 | 1 | 1 | 1 | 1 | 1 | 2 | 1 | — | 1 | — | 1 | 1 | 5 | 5 | 5 | 3 | 3 |
| ***NK6*** | 2 | 1 | — | 1 | 1 | 1 | 1 | 2 | 2 | 1 | 1 | 1 | 1 | 1 | 1 | 3 | 2 | 3 | 3 | 3 |
| ***NK7*** | — | 1 | 1 | — | 1 | — | — | — | — | 1 | 1 | 1 | — | 1 | 1 | — | — | — | — | — |
| ***Msx*** | 1 | 1 | 2 | 1 | 1 | 1 | 1 | 1 | 2 | 1 | 1 | 2 | 2 | 1 | 1 | 2 | 2 | 5 | 3 | 3 |
| ***Lbx*** | — | 1 | 1 | 1 | — | 1 | 1 | 1 | 1 | 1 | 1 | 1 | 1 | 2 | 1 | 1 | 1 | 3 | 2 | 2 |
| ***Tlx*** | 1 | 1 | 1 | 1 | — | 1 | 1 | 1 | 2 | 1 | 1 | 1 | 1 | 1 | 1 | 3 | 2 | 4 | 3 | 3 |
| ***NK2.1*** | — | ? | 1 | 1 | 1 | 1 | — | 1 | — | 1 | 1 | 1 | 1 | 1 | 1 | 4 | 1 | 3 | 2 | 2 |
| ***NK2.2*** | — | 5^?^ | 2 | 1 | 1 | 1 | — | 1 | 2 | 2 | 1 | 1 | 1 | 1 | 1 | 2 | 1 | 3 | 2 | 2 |
| ***Abox*** | — | — | — | ? | 1 | 1 | 1 | — | — | 1 | — | 1 | 1 | 1 | 1 | — | — | — | — | — |
| ***Ro*** | — | 1 | — | ? | 1 | 1 | 1 | — | 1 | 1 | — | 1 | 1 | 1 | 1 | — | — | — | — | — |
| ***Nedx*** | — | 2 | — | ? | — | 1 | 1 | 1 | — | 1 | — | 1 | 1 | 1 | 2 | — | — | — | — | — |
| ***vax*** | — | 2 | — | ? | 2 | — | — | 1 | — | 1 | — | — | — | — | 1 | 2 | 1 | 2 | 2 | 2 |
| ***Noto*** | — | 1 | — | ? | — | — | — | — | — | 1 | 1 | 1 | 1 | 1 | 1 | 1 | 2 | 1 | 1 | 1 |
| ***Emx*** | — | 2 | 2 | ? | 2 | 1 | 1 | 1 | 4 | 1 | 1 | 1 | 1 | 2 | 3 | 4 | 2 | 3 | 2 | 2 |
| ***BarH*** | 1 | — | 2 | ? | 2 | 2 | 3 | 1 | 2 | — | 2 | 1 | 1 | 2 | 1 | 2 | — | 3 | 2 | 2 |
| ***Bari*** | — | — | — | ? | — | — | — | 1 | 1 | 1 | 1 | 1 | 1 | 1 | 1 | — | — | — | — | — |
| ***Barx*** | — | — | — | ? | — | — | 2 | — | 1 | — | — | — | — | — | 1 | 2 | 1 | 2 | 2 | 2 |
| ***Hlx*** | — | 7 | — | ? | — | — | — | — | — | — | — | 1 | 1 | 1 | 1 | 1 | — | 1 | 1 | 1 |
| ***Dbx*** | — | — | — | ? | — | — | — | — | — | — | — | 1 | 1 | 1 | 1 | 2 | 2 | 3 | 2 | 2 |
| ***Bsx*** | — | — |  | ? | 1 | — | — | — | — | — | — | 1 | 1 | 1 | 1 | 1 | 1 | 1 | 1 | 1 |
| ***Hhex*** | 1 | 1 | 1 | ? | 1 | 1 | 1 | 1 | — | 1 | — | 1 | — | 1 | 1 | 2 | 1 | 1 | 1 | 1 |
| ***Nanog*** | — | — | — | ? | — | — | — | — | — | — | — | — | — | — | — | — | — | — | 3(2) | 12 (11) |
| ***Ventx*** | — | — | — | ? | — | — | — | — | — | — | — | — | — | — | 2 | 6 | 1 | 1 | — | 8 (7) |

^1^ [[1](#_ENREF_1)]; ^2^ [[2](#_ENREF_2), [3](#_ENREF_3)]; ^3^ [[4](#_ENREF_4)]; ^4^ [[5](#_ENREF_5)]; ^5^ [[6-8](#_ENREF_6)]; ^6^ [[9](#_ENREF_9)]

1. Zhong Y-f, Holland PWH: **HomeoDB2: functional expansion of a comparative homeobox gene database for evolutionary developmental biology.** *Evol Dev* 2011, **13:**567–568.

2. Saudemont A, Dray N, Hudry B, Le Gouar M, Vervoort M, Balavoine G: **Complementary striped expression patterns of NK homeobox genes during segment formation in the annelid *Platynereis*.** *Dev Biol* 2008, **317:**430–443.

3. Denes AS, Jékely G, Steinmetz PRH, Raible F, Snyman H, Prud'homme B, Ferrier DEK, Balavoine G, Arendt D: **Molecular architecture of annelid nerve cord supports common origin of nervous system centralization in Bilateria.** *Cell* 2007, **129:**277–288.

4. Chipman AD, Ferrier DEK, Brena C, Qu J, Hughes DST, Schröder R, Torres-Oliva M, Znassi N, Jiang H, Almeida FC, et al: **The first myriapod genome sequence reveals conservative arthropod gene content and genome organisation in the centipede *Strigamia maritima*.** *PLoS Biol* 2014, **12:**e1002005.

5. Hashimoto T, Horikawa DD, Saito Y, Kuwahara H, Kozuka-Hata H, Shin-I T, Minakuchi Y, Ohishi K, Motoyama A, Aizu T, et al: **Extremotolerant tardigrade genome and improved radiotolerance of human cultured cells by tardigrade-unique protein.** *Nat Commun* 2016, **7:**12808.

6. Hering L, Henze MJ, Kohler M, Bleidorn C, Leschke M, Nickel B, Meyer M, Kircher M, Sunnucks P, Mayer G: **Opsins in Onychophora (velvet worms) suggest a single origin and subsequent diversification of visual pigments in arthropods.** *Mol Biol Evol* 2012, **29**.

7. Hering L, Mayer G: **Analysis of the Opsin Repertoire in the Tardigrade *Hypsibius dujardini* Provides Insights into the Evolution of Opsin Genes in Panarthropoda.** *Genome Biol Evol* 2014, **6:**2380–2391.

8. Arakawa K, Yoshida Y, Tomita M: **Genome sequencing of a single tardigrade *Hypsibius dujardini* individual.** *Sci Data* 2016, **3:**160063.

9. Colbourne JK, Pfrender ME, Gilbert D, Thomas WK, Tucker A, Oakley TH, Tokishita S, Aerts A, Arnold GJ, Basu MK, et al: **The Ecoresponsive Genome of *Daphnia pulex*.** *Science (New York, NY)* 2011, **331:**555–561.
