# Supplementary figures and images for: Expression of NK cluster genes in the onychophoran Euperipatoides rowelli: implications for the evolution of NK family genes in nephrozoans
Source: EvoDevo. 2018 Jul 18;9:17. doi: 10.1186/s13227-018-0105-2 (PMC6050708; doi:10.1186/s13227-018-0105-2)

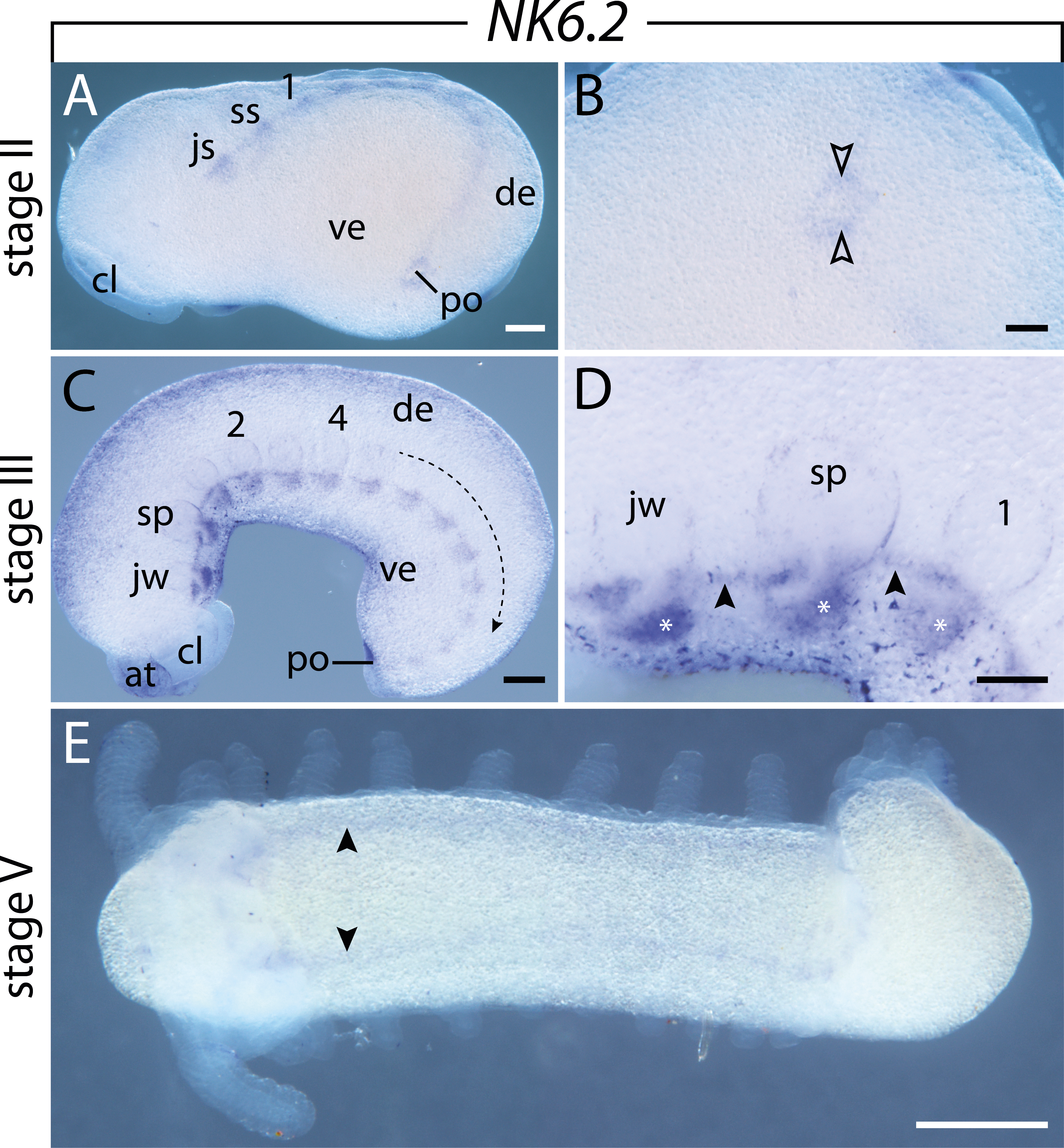

Supplement: Supplementary file 3 — Additional file 3. NK6.2 expression at consecutive developmental stages in embryos of the onychophoran E. rowelli. Developing limbs are numbered. Anterior is left in all images. A Stage II embryo in ventrolateral view. B Proctodeum of a stage II embryo in ventral view. Arrowheads indicate the expression around the proctodeum. C Stage III embryo in lateral view. Arrow indicates the decreasing intensity of the signal. D Developing jaw, slime papilla and first leg of a stage III embryo. Signals in the ventral ectodermal thickenings and ventral nerve cords in are indicated with asterisks and arrowheads, respectively. E Stage V embryo in ventral view. A weak expression is visible in the ventral nerve cords (arrowheads). Abbreviations: at, developing antenna; cl, cephalic lobe; de, dorsal extra-embryonic tissue; jw, developing jaw; js, jaw segment; po, proctodeum; sp, developing slime papilla; ss, slime papilla segment; ve, ventral extra-embryonic tissue. Scale bars: A, C: 200 µm; B, D: 100 µm: E: 500 µm. [file 13227_2018_105_MOESM3_ESM.tif]

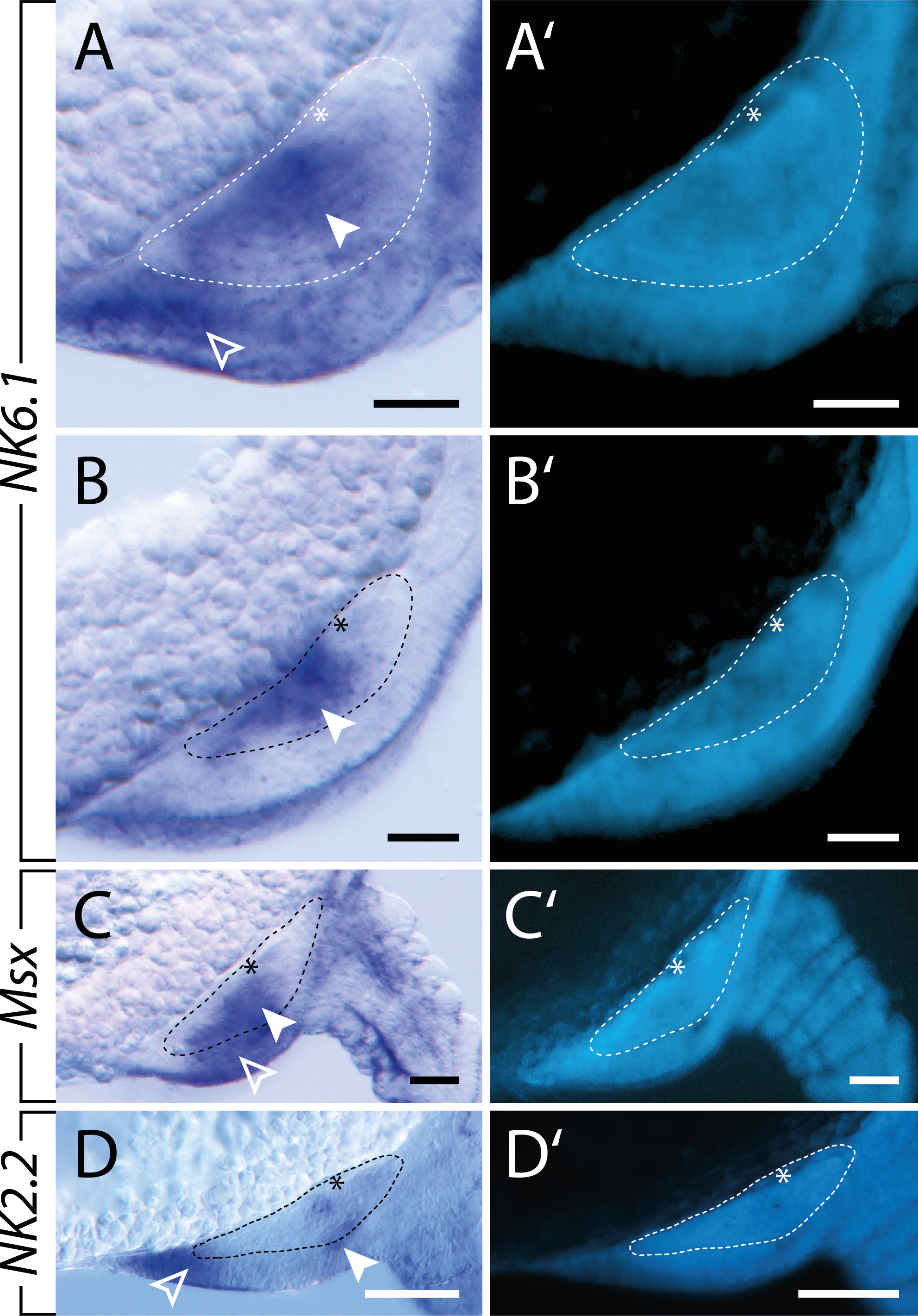

Supplement: Supplementary file 4 — Additional file 4. Cross sections of stage IV embryos of the onychophoran E. rowelli showing the expression of NK6.1 (A, A’, B, B’), Msx (C, C’) and NK2.2 (D, D’) in the ventral ectoderm (empty arrowheads) and developing nerve cords (filled arrowheads). Developing nerve cord is indicated with dashed lines, developing neuropil is indicated with asterisks. Scale bars: A–C’: 50 µm; D, D’: 100 µm. [file 13227_2018_105_MOESM4_ESM.tif]
